# Screening and Evaluation of Salt-Tolerant Wheat Germplasm Based on the Main Morphological Indices at the Germination and Seedling Stages

**DOI:** 10.3390/plants13223201

**Published:** 2024-11-14

**Authors:** Yunji Xu, Xuelian Weng, Liqiu Jiang, Yu Huang, Hao Wu, Kangjun Wang, Ke Li, Xiaoqian Guo, Guanglong Zhu, Guisheng Zhou

**Affiliations:** 1 Joint International Research Laboratory of Agriculture and Agri-Product Safety of the Ministry of Education of China, Yangzhou University, Yangzhou 225009, China; yunjixu@yzu.edu.cn (Y.X.); 18118277525@163.com (X.W.); liqiujiang1115@163.com (L.J.); 18552756918@163.com (Y.H.); w13964483386@163.com (H.W.); guoxqianqian@163.com (X.G.); g.zhu@yzu.edu.cn (G.Z.); 2 Lianyungang Academy of Agricultural Sciences, Lianyungang 222000, China; kjwang13@163.com; 3 Huaiyin Institute of Agricultural Sciences of the Xuhuai District of Jiangsu Province, Huaian 223001, China; lk2311586@163.com

**Keywords:** wheat (*Triticum aestivum* L.), salt tolerance, germination and seedling stages, morphological trait, multivariate statistical analysis

## Abstract

The successful screening and evaluation of salt-tolerant germplasm at the germination and seedling stages is of great importance for promoting the breeding of wheat varieties with salt tolerance. In this study, 70 wheat varieties bred in different regions were evaluated for salt tolerance through hydroponic exposure to different concentrations of salt. The relative water absorption, water absorption rate, dehiscence rate, germination rate, and germination index of seeds, and plant height, root length, stem diameter, and biomass of seedlings were determined at the germination and seedling stages of wheat, and the salt tolerance was identified and evaluated using multivariate statistical analysis. The germination ability and seedling growth potential of wheat germplasms decreased with the aggravation of salt stress. Based on the comprehensive salt tolerance index at the germination stage, our study identified 35 varieties to be salt-tolerant. There were nine varieties further screened for having strong salt tolerance according to the comprehensive salt tolerance index at the germination and seedling stages. SN41, Emam, YN301, and JM262 were superior in salt-tolerance, and YM39, LM30, JM60, YN999, and SD29 were salt-tolerant. Our study suggests that the biomass of seedlings can be used as a key parameter for assessing wheat germplasm’s ability to withstand salt. Our results can provide some basic materials for cultivating new germplasm with salt tolerance and excavating the related genes of wheat.

## 1. Introduction

In the modern world, a lot of arable land has been occupied due to the quick growth of urbanization and industrialization [1,2,3,4]. Accordingly, one of the most important foundations for ensuring global food security is the development of reserve or marginal land resources [5]. The development and enhancement of coastal areas have garnered increasing attention as a significant component of marginal soil [6,7,8,9,10]. The most cost-effective and efficient method of making sensible use of coastal land and boosting agricultural productivity is the successful cultivation of salt-tolerant crops [11,12,13]. According to studies, crops grown in salty soil frequently have a lower rate of germination and slower seedling growth, which has a major impact on crop production, quality, and financial gain [14,15,16,17]. Therefore, the screening and breeding of crops with significant salt tolerance is of great significance to the development of agricultural production and the raising of farmers’ incomes in coastal areas.

Wheat (*Triticum aestivum* L.) is one of the three major food crops around the globe, feeding about 35~40% of the world’s population as a staple food [18]. Among cereal crops, wheat is a medium salt-tolerant crop, showing more tolerance than rice but less tolerance than sorghum and barley [19,20], and the salt tolerance of wheat cultivars also varies widely [21,22,23]. Additionally, wheat is a self-pollinating crop, and wheat varieties are actually inbred lines with a high level of genetic and phenotypic uniformity [24]. Worldwide, extensive efforts have been made to date to screen, evaluate, identify, and breed salt-tolerant wheat varieties (lines) [15,23,25]. The key to successful salt-tolerance breeding in wheat is the screening and assessment of salt-tolerant genetic resources. Studies have shown that plant establishment and yield potential are significantly influenced by rapid seed germination and excellent seedling growth [26,27,28,29]. Thus, it is well acknowledged that the germination and seedling stages are suitable timepoints to screen germplasm collections for salt [21,30,31]. However, the majority of researchers have individually concentrated on screening and assessing wheat’s tolerance to salt during the germination or seedling stage [22,23,25]. Fewer research studies have been conducted at the germination and seedling stages concurrently.

At the germination stage of wheat, germination ability is commonly assessed using the seed water absorption rate, germination rate, and germination index. The growth characteristics of wheat seedlings, such as plant height, root length, stem diameter, and biomass, are also considered significant indicators for estimating salt tolerance [32,33]. In addition to the morphological parameters mentioned above, numerous physiological traits, such as proline, soluble sugars, malondialdehyde, the ratio of Na^+^/K^+^, and antioxidant enzyme activities in wheat seedlings, have been recognized as appropriate markers for identifying salt-tolerant varieties [34,35]. In the meantime, wheat’s salt tolerance is often assessed by computing the salt tolerance index of each morphological or physiological parameter, as well as the comprehensive salt tolerance index of all measured parameters [22,36]. Compared with physiological parameters, morphological traits are much easier to observe and quantify. Most importantly, the improvement in morphological or agronomic traits is always the central work and main goal in wheat breeding [37,38]. Because of this, screening salt-tolerant wheat germplasm using morphological indices is more practically significant.

Furthermore, growing China’s wheat output is crucial to guaranteeing global food security because it is the agricultural nation that produces the most wheat. Therefore, this study was mainly conducted to identify salt-tolerant varieties from wheat germplasm resources bred in China. The workflow began with screening 35 salt-tolerant germplasms from 70 tested wheat varieties based on the main morphological indices at the germination stage. Next, we performed a comprehensive analysis of salt tolerance during the germination and seedling stages on a subset of 35 varieties with increased salt tolerance in order to identify material with superior salt tolerance. Identifying certain elite wheat germplasms with high salt tolerance and presenting the effective evaluation index were our ultimate goals.

## 2. Results

### 2.1. Descriptive Analysis of Salt Tolerance in Wheat at Germination Stage

Our findings indicate that there are notable variations in the germination characteristics of 70 wheat varieties, with coefficients of variation for relative water absorption (RWA), water absorption rate (WAR), dehiscence rate (DR), germination rate (GR), and germination index (GI) of wheat seeds reaching as high as 31.9~39.5%, 31.8~35.2%, 16.0~44.9%, 18.9~53.4%, and 20.9~31.3%, respectively (Table 1). Table 1 demonstrates that the two salt treatment groups’ mean, minimum, and maximum values of RWA, WAR, DR, GR, and GI were all lower than those of the control (CK) group. The reduction rate was most pronounced under 180 mM NaCl treatment. According to these findings, wheat seed germination was inhibited by salt treatment, and the higher the concentration of salt stress, the more severe the inhibition effect. Furthermore, with CK treatment, the coefficient of variation in DR, GR, and GI was 16.0~20.9%, whereas under 140 mM NaCl and 180 mM NaCl treatments, the coefficients of variation were as high as 24.9~30.9% and 31.3~53.4%, respectively (Table 1). According to these findings, there were significant differences in the germination stage of salt tolerance across 70 distinct wheat varieties.

### 2.2. Membership Function Analysis of the Comprehensive Salt Tolerance Index of Wheat at the Germination Stage

Table S1 shows each measuring parameter’s salt tolerance index for 70 wheat varieties at the germination stage under 140 mM and 180 mM NaCl treatments. The salt tolerance index varied greatly with wheat germplasms at the germination stage (Table S1). In general, the germination stage salt tolerance index values under the 140 mM NaCl treatment were greater than those under the 180 mM NaCl treatment (Table S1).

With 70 varieties, the membership function analysis revealed that the D_1_ values (comprehensive salt tolerance index) ranged widely, from 0.01 to 0.83 (Table 2). The top 35 wheat varieties with a D_1_ value higher than 0.4 were initially screened as salt-tolerant wheat germplasms at the germination stage based on the comprehensive salt tolerance index of various parameters (Table 2). JM262, SD29, XM44, JM60, ZM1860, YN301, JM54, YN999, Emam, STM1, YM39, LM30, YN1212, SN41, XM35, YM23, HM20, ZM136, HM168, YM33, ZM15, YJM586, HM35, NM21, JM52, RH520, WM206, ZYGK1, LM1936, WY16, YM16, LM10, HY66, TM178, and YM34 were among them (Table 2). In the following, the salt tolerance of these 35 wheat varieties is thoroughly assessed during the germination and seedling stages.

### 2.3. Changes in the Main Morphological Indices of Wheat Seedlings Under Different Salt Treatments and the Salt Tolerance Index

**Figure 1:**
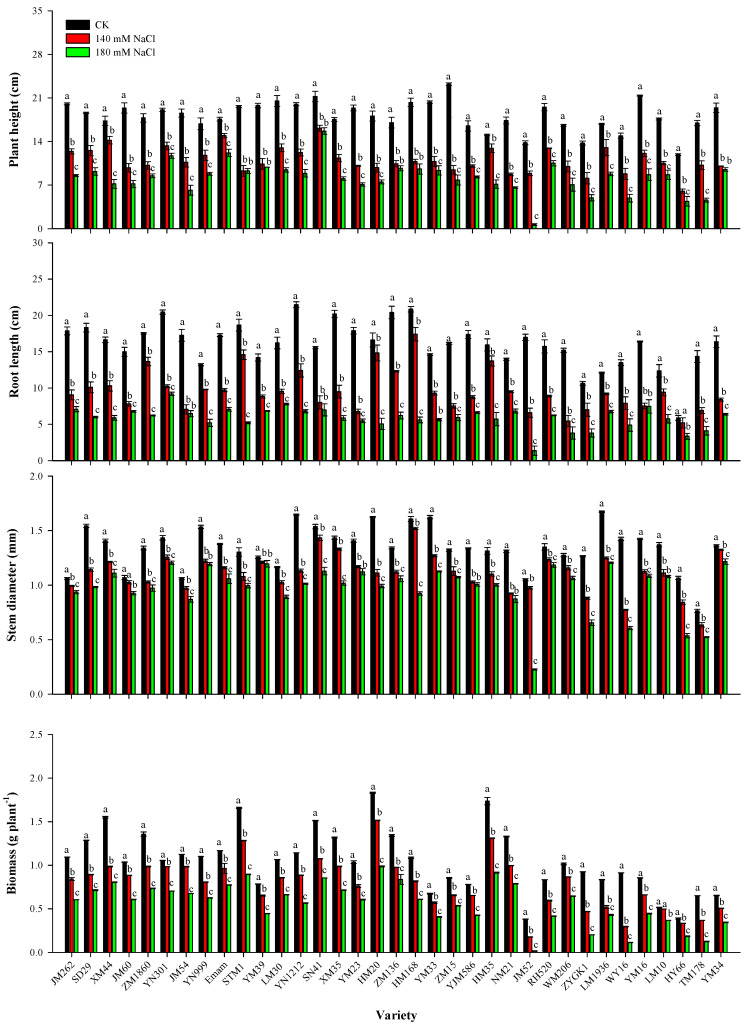
Changes in the main morphological traits of 35 wheat varieties at the seedling stage under control conditions (CK), 140 mM NaCl, and 180 mM NaCl. Data are expressed as the mean ± standard error (*n* = 6). Different letters above the bars indicate the least significance at the *p* < 0.05 level within each wheat variety.

Figure 1 illustrates how the above 35 wheat varieties changed in terms of plant height (PH), root length (RL), stem diameter (SD), and biomass (BM) during the seedling stage when exposed to various salt treatments. With 35 distinct wheat varieties, PH, RL, SD, and BM differed under the control (CK, no salt stress) treatment (Figure 1). The PH, RL, SD, and BM of each wheat variety at the seedling stage were significantly decreased by the two salt treatments (140 mM NaCl and 180 mM NaCl) in comparison to the CK treatment; the loss was more pronounced under the 180 mM NaCl treatment (Figure 1). According to calculations, the 140 mM NaCl treatment reduced the PH, RL, SD, and BM of 35 wheat varieties by 14.3~59.3%, 10.8~64.1%, 13.7~45.7%, and 3.6~67.6%, respectively, in comparison to the CK treatment, whereas 180 mM NaCl decreased these by 26.2~95.2%, 43.1~91.9%, 4.8~78.4%, and 28.6~96.1%, respectively (Figure 1).

The salt tolerance index for each parameter in the 35 wheat varieties at the seedling stage under two salt treatments is displayed in Table S2. Different wheat germplasms also showed significant differences in the salt tolerance index at the seedling stage (Table S2). When exposed to 140 mM NaCl at the seedling stage, the salt tolerance indices were essentially higher than when exposed to 180 mM NaCl (Table S2).

### 2.4. Correlation Between Salt Tolerance Index of Various Morphological Indices and Comprehensive Salt Tolerance Index at the Germination and Seedling Stages

Table 3 displays the membership function analysis’s comprehensive salt tolerance index (D_2_ value) for nine morphological parameters that were assessed during the germination and seedling phases of 35 different wheat varieties. With 35 varieties, the D_2_ value likewise varied significantly, ranging from 0.22 to 0.75 (Table 3).

**Figure 2:**
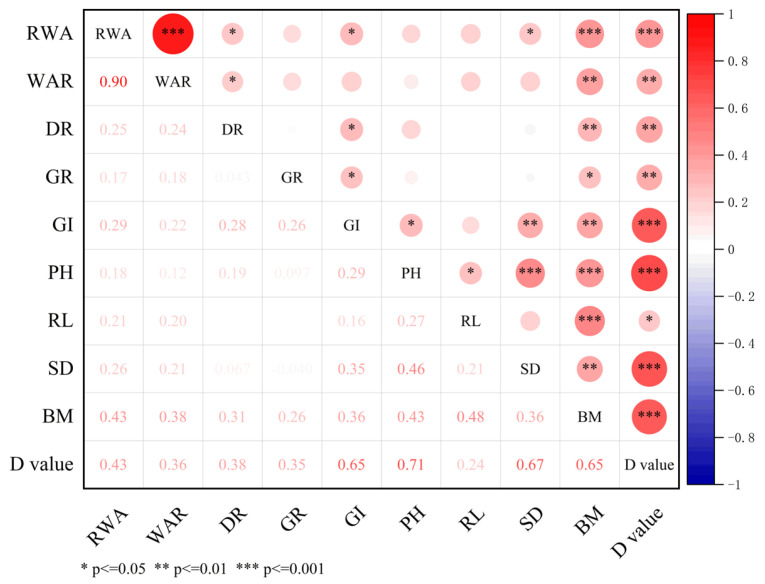
Correlation between the salt tolerance index of various morphological indices and comprehensive salt tolerance indices (D_2_ value) at the germination and seedling stages of 35 wheat varieties. RWA, relative water absorption; WAR, seed water absorption rate; DR, seed dehiscence rate; GR, seed germination rate; GI, seed germination index; PH, plant height; RL, root length; SD, stem diameter; BM, biomass.

The correlation analysis revealed a considerable or highly positive correlation between the salt tolerance index of each measured parameter and the comprehensive salt tolerance index (*r* = 0.24 *~0.71 ***, Figure 2). In contrast, the comprehensive salt tolerance index had higher correlation coefficients (*r* = 0.43 ***~0.71 ***) with the salt tolerance index of RWA, GI, PH, SD, and BM (Figure 2). The findings showed that the following factors significantly influenced wheat’s ability to withstand salt: RWA, GI, PH, SD, and BM.

### 2.5. Principal Component Analysis of Each Salt Tolerance Index at the Germination and Seedling Stages of Wheat

**Figure 3:**
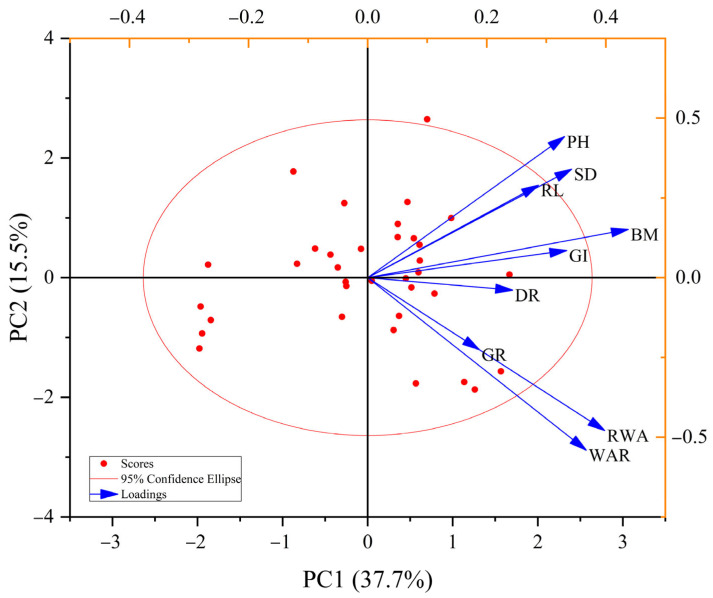
Principal component analysis of the salt tolerance index for each morphological parameter at the germination and seedling stages of 35 wheat varieties. RWA, relative water absorption; WAR, seed water absorption rate; DR, seed dehiscence rate; GR, seed germination rate; GI, seed germination index; PH, plant height; RL, root length; SD, stem diameter; BM, biomass.

With the cumulative contribution rate reaching 80% as the threshold for the extraction of principal components, nine morphological indices could be simplified into five independent components (PC1~PC5), and the cumulative contribution rate was 84.83% (Table 4). The first principal component (PC1) accounted for 37.61%, with high factor loadings for BM (0.81), RWA (0.73), WAR (0.67), SD (0.63), GI (0.62), and PH (0.61) (Table 4); the second principal component (PC2) accounted for 15.51%, in which the factor loadings of WAR (−0.57), RWA (−0.64), and PH (0.52) were the highest (Table 4). The factor loadings for the third principal component (PC3) and third principal component (PC4) were highest for GR and DR, respectively. The fifth principal component had the highest factor loading for RL (Table 4). In addition, it is evident from Figure 3 that the salt tolerance indices of GI, PH, RL, SD, and BM were all clustered in the first quadrant, positively relating to both PC1 and PC2. Among these, their contributions to PC1 presented a tendency of BM > SD > GI > PH > RL (Figure 3). Nevertheless, the salt tolerance indices of RWA and WAR were scattered in the fourth quadrant, significantly negatively relating to PC2 (Figure 3). Collectively, all of the morphological parameters were associated with wheat’s ability to withstand salt, and the BM of seedlings was suggested as a key parameter for assessing the germplasm’s ability to withstand salt stress.

### 2.6. Cluster Analysis of the Comprehensive Salt Tolerance Index of Various Morphological Parameters in Wheat at the Germination and Seedling Stages

**Figure 4:**
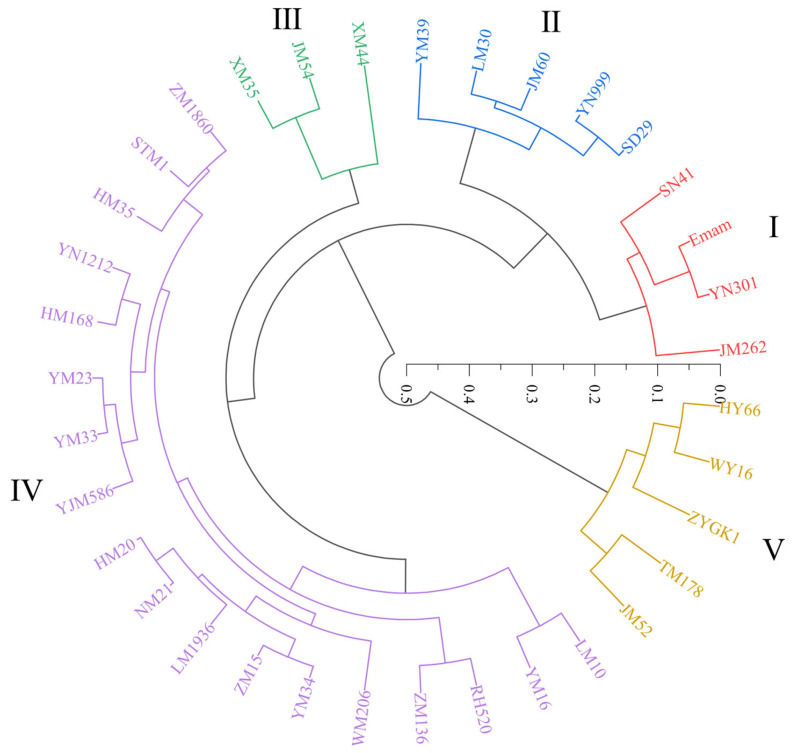
Cluster analysis of comprehensive salt tolerance index of 35 wheat varieties at the germination and seedling stages. Those with similar squared Euclidean distances were divided into one cluster. Different colors represent a cluster. I, superior salt-tolerant type; II, salt-tolerant type; III, medium salt-tolerant type; IV, salt-sensitive type; V, extremely salt-sensitive type.

When the Euclidean square distance was set at 0.17 (Figure 4), the 35 wheat varieties mentioned above were categorized into five classes based on the comprehensive salt tolerance index of various measured parameters at the germination and seedling stages (Table 3, Table S2). The first class was superior in its salt tolerance, including SN41, Emam, YN301, and JM262; the second was a salt-tolerant type, with YM39, LM30, JM60, YN999, and SD29; the third was a medium salt-tolerant type containing XM35, JM54, and XM44; the fourth was a salt-sensitive type, with 18 varieties such as LM10, YM16, RH520, and ZM136; and the fifth was extremely salt-sensitive, consisting of HY66, WY16, ZYGK1, TM178, and JM52 (Figure 4).

Strongly salt-tolerant wheat varieties made up the first and second classes of the cluster analysis above (Figure 4). As a result, four wheat varieties were further chosen as wheat germplasms with superior salt tolerance: SN41, Emam, YN301, and JM262. These were followed by YM39, LM30, JM60, YN999, and SD29 (Figure 4).

## 3. Discussion

Crops’ phenotypic characteristics are significantly impacted by salinity stress, and these responses are typically manifested at the molecular, cellular, metabolic, physiological, and morphological levels [39,40]. When compared to other responses, morphological changes are the most obvious. Given the above, a number of morphological traits, such as seed germination rate [41], root length [42], and plant biomass [43,44], have been deemed appropriate evaluation indices for identifying salt-tolerant crops. In this study, we consistently monitored germination and seedling growth to screen and identify wheat varieties with increased salt tolerance. A popular technique for screening wheat germplasms for salt tolerance is hydroponics, which simulates salt stress using either one salt type (NaCl) or two salt types (NaCl, Na_2_SO_4_, or Na_2_CO_3_) [41,45,46,47]. Wheat seeds and seedlings were treated in the current study using two salt concentrations (140 mM and 180 mM NaCl), which are regarded as inducing high salt stress [36,41,45]. According to our findings, salt treatment significantly hindered the water uptake and germination of wheat seeds (Table 1) and decreased the PH, RL, SD, and BM of wheat seedlings (Figure 1). The 180 mM NaCl treatment had a more negative effect on seed germination and seedling growth than the 140 mM NaCl treatment (Table 1, Figure 1). These findings are congruent with previous reports [41,45].

Wheat’s ability to withstand salt is a complex characteristic that is regulated by several genes and impacted by both environmental and genetic variables [34,48]. It is challenging to utilize a single metric to accurately depict wheat’s salt tolerance because plants react to salt stress through a range of morphological and physiological pathways [35,49]. Numerous domestic and international studies have been conducted on the screening and assessment of agricultural germplasm resources for salt tolerance [22,29,30,50]. Crops’ tolerance to salt [51], drought [52,53], and cold [54] have so far been effectively assessed using a variety of multivariate statistical techniques, such as membership function analysis, principal component analysis, and cluster analysis. The salt tolerance of rice [55], maize [56], tomato [57], and celery [50] has often been estimated using these multivariate statistical techniques. In wheat, Peng et al. [51] selected 18 materials with high salt tolerance from 321 wheat materials depending on the comprehensive salt tolerance evaluation (D value), which was calculated by combining principal component analysis and membership function values. By using cluster analysis on the principal component analysis results, Li et al. [58] eliminated 10 saline–alkali-tolerant materials from 283 wheat germplasms. Through cluster analysis, Gao et al. [36] divided 236 wheat germplasms into five groups based on the comprehensive salt tolerance index (D value) at the seedling stage. In the present study, we adopted the method of Gao et al. [36] to evaluate the salt tolerance of 70 wheat varieties. We further screened superior salt-tolerant materials using the principal component analysis (Table 4) and cluster analysis (Figure 4). Furthermore, it should be mentioned that JM262 was ultimately found to be among the germplasms in our study that are incredibly salt-tolerant (Figure 4). In a previous study, JM262 was consistently reported to be a highly salt-tolerant germplasm [59]. This identical outcome may serve as an indirect demonstration of the precision and soundness of the screening strategy used in this investigation.

The comprehensive salt tolerance evaluation approach is frequently used to assess the salt tolerance of various crop varieties, as previously mentioned. However, because of this high workload, it is not practical for the quick identification of vast amounts of agricultural materials [36,60]. As a result, screening some appropriate markers that can rapidly indicate wheat’s salt tolerance has become essential. Commonly used morphological evaluation indexes for wheat salt tolerance vary depending on the crop, variety, or stage of growth [30,31,35,61,62,63]. At the germination stage, embryo root length, bud length, germination rate, budding potential, etc., were reported as key parameters [30,35]. Plant height, root length, the number of roots, the fresh and dry weight of roots, the fresh and dry weight of stems and leaves, the salt tolerance coefficient, etc., were considered important parameters for salt tolerance at the seedling stage [31,61]. When conducting field or pot experiments, the entire growth period of plants, biomass, plant height, population quality, yield, and its components, etc., are usually used for salt tolerance evaluation [62,63]. In our study, correlation analysis showed the salt tolerance indexes for RWA, GI, PH, SD, and BM to have significantly positive relations with the comprehensive salt tolerance index of 35 varieties (Figure 2). Based on the results of PCA, the salt tolerance indexes of RWA, GI, PH, SD, and BM also contributed more to PC1, with BM making the largest contribution (Table 4, Figure 3). Hereby, these five characters, including RWA, GI, PH, SD, and BM, could be used as important parameters for estimating salt tolerance, and the salt tolerance index of BM (biomass) was the best option. Similarly, Ti et al. [64] earlier proposed that biomass could be the key parameter for evaluating wheat salinity tolerance in the Hertao Irrigation District of China. According to Griffa et al. [65], seedling biomass and its constituent parts may serve as selection criteria for enhancing salt tolerance in Buffel grass genotypes. Given this, we firmly think that wheat seedling biomass can serve as a significant parameter of salt tolerance.

## 4. Materials and Methods

### 4.1. Experimental Materials

The study was carried out from October 2021 to February 2022 in the Joint International Research Laboratory of Agriculture and Agri-Product Safety of the Ministry of Education of China, Yangzhou University (32°30′ N, 119°43′ E), Jiangsu Province, China. In this study, seeds of 70 wheat varieties from various breeding regions were employed as test materials. The 68 varieties were bred in China, with 41 from Jiangsu Province, 9 from Anhui Province, 9 from Shandong Province, 1 from Hebei Province, 5 from Henan Province, 2 from Shaanxi Province, and 1 from Heilongjiang Province (Table 5). The other two wheat lines were from Africa’s Northern Sudan (Table 5). Table S3 displays the primary agronomic characteristics of wheat varieties.

### 4.2. Salt Treatment

From each wheat variety, 180 plump and uniformly sized seeds were chosen to be sterilized for 10 min using a 1.5% sodium hypochlorite solution after soaking in distilled water for 2 min; they were then rinsed with distilled water 2~3 times before being dried with sterile filter paper. Subsequently, seeds were put in a 9 cm diameter Petri dish lined with two layers of filter paper. The seeds were then subjected to three distinct salt treatments: (1) distilled water or a control (CK); (2) 140 mM NaCl or 140 mM sodium chloride solution; and (3) 180 mM NaCl or 180 mM sodium chloride solution. In total, 10 mL of solution and twenty healthy seeds were placed in each Petri dish, and each treatment was repeated three times. To reduce evaporation, Petri dishes were covered with lids. For the germination test, these Petri dishes were placed in an incubator at 25 °C with alternating light and dark conditions for 12 h and a relative humidity of 55–60%. To keep the salt solution consistent throughout this time, the seeds in each Petri dish were moved to a fresh dish every 6 h. For 3 days, the seeds that had germinated were moved to a 96 mm sieve plate with the bottom removed. They were then placed inside a black frame with the appropriate culture media (salt solution or distilled water) and Hoagland nutrient solution. To maintain a steady salt solution, the culture medium and nutrition solution were changed every day. The pertinent wheat seedling indexes were sampled one week later (early seedling stage) in order to determine and compute the samples.

### 4.3. Measurement Items and Methods

The seed germination indices of 70 test wheat varieties, including RWA, WAR, DR, GR, and GI of seeds, were initially examined to identify 35 varieties with increased salt tolerance. Subsequently, seedling growth parameters of 35 varieties, containing PH, RL, SD, and BM, were further measured and combined with seed germination indices to comprehensively evaluate wheat’s salt tolerance.

#### 4.3.1. Seed Germination Indices

To determine the RWA and WAR of the seeds, five sample wheat seeds were chosen at random from each Petri dish, dried with absorbent paper, and weighed. The weight of water absorbed as a percentage of the seed’s initial weight is known as the RWA (%). The amount of water absorbed per unit of time is known as the WAR (mg h^−1^). Every twelve hours, the number of seeds that dehisced and germinated was counted individually. The DR (%) was determined on the third day of incubation by calculating the percentage of the germination number and the number of seeds that had dehisced, accounting for the total number of seeds. We considered seeds to be germinated when the bud length started to surpass 50% of the root length. Two days after transfer, the GR (%) was calculated by dividing the number of seeds that germinated that day by the total number. The GI was calculated as follows: GI = Σ (number of seeds germinated on the day/days of germination).

#### 4.3.2. Seedling Growth Parameters

At the seedling stage, PH, RL, SD, and BM were measured with six repetitions. From each treatment, six wheat seedlings with comparatively steady growth were removed and put on sterile filter paper. Using a ruler, the PH was determined by measuring the distance between the base of the roots and the tip of the longest leaf. Using a ruler, the RL was measured from the primary root’s growing position to the tip. The SD was measured at the stem base using a vernier caliper. Two seedlings were used as a replicate, and twelve fresh wheat seedlings were sampled and dried at 80 °C to a constant weight to determine the final BM.

### 4.4. Data Analysis

Microsoft Excel 2019 was used to determine each morphological parameter’s mean value, and Statistix 9 software was used to conduct the analysis of variance (ANOVA). The SPSS 27 program was utilized to perform principal component analysis (PCA) and membership function analysis based on the salt tolerance index of each morphological parameter of wheat germplasm. The SigmaPlot 10.0 was applied for graphing, and Origin 2024 software was used to draw the cluster analysis tree according to the comprehensive salt tolerance index, and the correlation analysis heat maps were graphed in the website chiplot.online. Before performing ANOVA, the Shapiro–Wilk (S-W) test was used to check the data normality, and the data conformed to a normal distribution.

During data analysis, each morphological parameter value was first translated into a salt tolerance index [36] for each parameter. The formula for calculating the salt tolerance index is as follows: salt tolerance index = (the value under the NaCl treatment/the value under the CK) × (the value under the NaCl treatment/the mean value of all germplasms under the NaCl treatment). Next, the salt tolerance indexes of various measured parameters were combined together to calculate the weighted membership function value (D value) using the membership function method. In this study, we designated the D value as the comprehensive salt tolerance index to evaluate salt tolerance.

The membership function was computed as previously described [36,66]. At the start, the principal component values (*X*) of the salt tolerance index of various morphological parameters were obtained from PCA. The membership function value (U) was calculated using the following: $U\left(X_{j}\right)={(X}_{j}-X_{min})/(X_{max}-X_{min}),\;j=1,2,3,\ldots ,n$. Here, *X_j_* represents the j-th principal component value, and *X_max_* and *X_min_* donate the maximum and minimum principal component value, respectively. The weighted value (*W*) was computed as follows: $W_{j}=P_{j}/\sum_{j=1}^{n}P_{j},\;j=1,2,3,\ldots ,n.$ Here, *W_j_* represents the weight assigned to the j-th principal component value within the entire set of values, and *P_j_* denotes the contribution rate of the j-th principal component value, which was also calculated by PCA. Finally, the D value was determined according to the following formula:$D=\sum_{j=1}^{n}\left[U(X_{j}\right){\times W}_{j}],\;j=1,2,3,\ldots ,n.$ In our study, the comprehensive salt tolerance index at the germination stage was designated as the D_1_ value, which, at the germination and seedling stages, was named the D_2_ value.

## 5. Conclusions

The seed germination capacity and seedling growth potential of wheat germplasms decreased with the increase in salt stress. SN41, Emam, YN301, and JM262 were superior in salt-tolerance, and YM39, LM30, JM60, YN999, and SD29 were salt-tolerant. The biomass of wheat seedlings was significantly and positively correlated with the comprehensive salt tolerance index of the main morphological indices, which could be used as an important parameter to evaluate salt tolerance. This study can provide references for the rapid identification and evaluation of salt tolerance in wheat.

## Figures and Tables

**Table 1:** Descriptive statistical analysis of main morphological indices of 70 wheat varieties at the germination stage under distinct salt treatments.

Treatment	Parameter	RWA /%	WAR /(mg h^−1^)	DR /%	GR /%	GI
CK	Minimum	47.0	29.1	30.0	26.7	18.5
	Maximum	205.5	111.6	100.0	100.0	69.3
	Mean	102.5	58.7	86.3	81.6	54.0
	Standard deviation	36.2	18.7	13.8	15.4	11.3
	Coefficient of variation/%	35.3	31.8	16.0	18.9	20.9
140 mM NaCl	Minimum	35.9	21.0	18.3	13.3	14.7
	Maximum	150.1	80.3	98.3	91.7	64.3
	Mean	75.5	43.0	68.3	59.9	45.9
	Standard deviation	24.1	12.7	21.1	16.4	11.4
	Coefficient of variation/%	31.9	29.5	30.9	27.4	24.9
180 mM NaCl	Minimum	24.0	12.8	0.0	0.0	10.7
	Maximum	148.0	74.3	95.0	81.7	60.7
	Mean	60.4	34.6	57.2	39.1	39.6
	Standard deviation	23.9	12.2	25.7	20.9	12.4
	Coefficient of variation/%	39.5	35.2	44.9	53.4	31.3

RWA, relative water absorption; WAR, seed water absorption rate; DR, seed dehiscence rate; GR, seed germination rate; GI, seed germination index.

**Table 2:** Salt tolerance index of each parameter and the comprehensive salt tolerance index of 70 wheat varieties at the germination stage under salt stress.

Variety (Line)	RWA ^#^	WAR	DR	GR	GI	Principal Component Value		Membership Function Value		Comprehensive Salt Tolerance Index * (D_1_ Value)	Ranking
						X_1_	X_2_	U_1_	U_2_		
JM262	0.72	0.90	1.38	1.46	1.35	1.62	1.61	0.82	0.85	0.83	1
SD29	1.40	1.35	1.38	1.00	1.18	2.26	−1.59	0.99	0.08	0.80	2
XM44	1.61	1.25	1.31	1.04	1.12	2.30	−1.91	1.00	0.00	0.79	3
JM60	0.73	0.77	1.50	1.25	1.29	1.37	1.72	0.76	0.88	0.78	4
ZM1860	1.35	1.32	1.22	1.17	0.93	1.99	−1.79	0.92	0.03	0.73	5
YN301	1.25	1.17	1.29	0.80	1.16	1.72	−1.17	0.85	0.18	0.71	6
JM54	1.22	1.16	1.50	0.94	0.88	1.66	−1.15	0.83	0.18	0.70	7
YN999	0.92	0.89	1.19	1.05	1.11	1.20	0.26	0.71	0.53	0.67	8
Emam	0.93	0.73	1.09	1.24	1.07	1.09	0.64	0.68	0.62	0.67	9
STM1	0.80	0.67	1.27	1.00	1.14	0.90	1.11	0.63	0.73	0.65	10
YM39	0.89	0.86	1.00	0.85	1.33	1.08	0.33	0.68	0.54	0.65	11
LM30	0.82	0.69	1.03	0.94	1.25	0.85	0.88	0.62	0.68	0.63	12
YN1212	0.95	1.14	1.08	0.96	0.98	1.24	−0.79	0.72	0.27	0.63	13
SN41	0.47	0.52	1.50	1.03	1.01	0.47	2.22	0.52	1.00	0.62	14
XM35	1.04	0.97	0.83	1.09	1.02	1.14	−0.65	0.70	0.31	0.61	15
YM23	0.76	0.75	1.17	0.81	1.12	0.73	0.68	0.59	0.63	0.60	16
HM20	0.80	0.93	1.20	0.76	1.04	0.86	0.03	0.63	0.47	0.59	17
ZM136	1.01	0.86	1.03	0.98	0.93	0.96	−0.36	0.65	0.38	0.59	18
HM168	0.75	0.74	1.09	1.01	1.00	0.70	0.66	0.58	0.62	0.59	19
YM33	0.94	0.87	0.91	0.75	1.19	0.89	−0.20	0.63	0.42	0.59	20
ZM15	0.80	0.93	0.77	1.01	1.02	0.78	−0.19	0.60	0.42	0.56	21
YJM586	0.74	0.72	0.75	0.96	1.16	0.58	0.58	0.55	0.60	0.56	22
HM35	0.64	0.76	1.10	0.91	0.97	0.53	0.71	0.54	0.64	0.56	23
NM21	0.79	0.81	0.48	1.13	0.95	0.50	−0.16	0.53	0.42	0.51	24
JM52	0.55	0.68	1.22	0.82	0.82	0.24	0.90	0.46	0.68	0.51	25
RH520	0.73	0.94	0.65	0.94	0.94	0.53	−0.38	0.54	0.37	0.50	26
WM206	0.63	0.65	1.22	0.60	0.91	0.21	0.72	0.45	0.64	0.49	27
ZYGK1	0.41	0.34	0.94	0.94	0.99	−0.17	2.09	0.35	0.97	0.48	28
LM1936	0.70	0.81	1.11	0.37	0.94	0.23	−0.10	0.46	0.44	0.46	29
WY16	0.50	0.60	0.99	0.76	0.85	−0.04	0.94	0.39	0.69	0.45	30
YM16	0.68	0.76	1.17	0.72	0.57	0.13	−0.09	0.43	0.44	0.43	31
LM10	0.53	0.34	1.05	0.61	0.93	−0.28	1.53	0.33	0.83	0.43	32
HY66	0.52	0.61	0.58	1.29	0.63	−0.06	0.65	0.38	0.62	0.43	33
TM178	0.67	0.66	0.98	0.62	0.80	0.04	0.22	0.41	0.52	0.43	34
YM34	0.87	0.82	0.39	0.76	0.94	0.27	−0.83	0.47	0.26	0.43	35
YM25	0.75	0.64	0.41	0.57	0.97	−0.13	−0.27	0.37	0.40	0.37	36
LM1738	0.49	0.49	0.41	0.59	1.09	−0.41	0.83	0.29	0.66	0.37	37
LM186	0.96	0.93	0.90	0.21	0.72	0.18	−1.63	0.45	0.07	0.37	38
YFM4	0.79	0.78	0.64	0.52	0.77	−0.02	−0.80	0.39	0.27	0.37	39
JM379	0.47	0.50	1.17	0.36	0.73	−0.45	0.85	0.28	0.67	0.36	40
LMG1302	0.41	0.35	1.13	0.50	0.70	−0.61	1.39	0.24	0.80	0.36	41
ZX998	0.62	0.58	0.83	0.61	0.65	−0.32	0.13	0.32	0.49	0.35	42
WL169	0.66	0.65	1.03	0.28	0.69	−0.29	−0.18	0.32	0.42	0.34	43
LM21137	0.61	0.64	0.43	0.54	0.91	−0.32	−0.08	0.31	0.44	0.34	44
WM203	0.74	0.76	0.36	0.60	0.82	−0.15	−0.78	0.36	0.27	0.34	45
LM1901	0.65	0.72	0.86	0.33	0.73	−0.26	−0.39	0.33	0.37	0.34	46
XSJ999	0.32	0.35	0.57	0.99	0.65	−0.72	1.41	0.21	0.80	0.34	47
RH502	0.35	0.32	0.45	0.78	0.79	−0.82	1.30	0.18	0.78	0.31	48
HM17	0.25	0.20	0.82	0.54	0.70	−1.07	1.80	0.12	0.90	0.28	49
SM132	0.42	0.40	0.83	0.22	0.75	−0.88	0.74	0.17	0.64	0.27	50
LM2193	0.68	0.60	0.54	0.56	0.50	−0.57	−0.61	0.25	0.31	0.26	51
HM16	0.57	0.55	0.54	0.32	0.71	−0.72	−0.18	0.21	0.42	0.25	52
HY1722	0.49	0.58	0.50	0.50	0.61	−0.74	−0.10	0.20	0.44	0.25	53
Elnilein	0.56	0.48	0.73	0.24	0.64	−0.80	0.01	0.19	0.47	0.25	54
HM2892	0.34	0.29	0.50	0.25	0.82	−1.18	0.97	0.09	0.70	0.22	55
LM1825	0.49	0.57	0.40	0.30	0.63	−0.94	−0.35	0.15	0.38	0.20	56
HM29262	0.46	0.47	0.65	0.08	0.67	−1.04	0.05	0.13	0.48	0.20	57
RH116	0.42	0.44	0.61	0.21	0.62	−1.08	0.22	0.11	0.52	0.20	58
LM231	0.63	0.65	0.21	0.17	0.68	−0.88	−1.08	0.17	0.20	0.18	59
LM1905	0.54	0.45	0.32	0.41	0.49	−1.10	−0.35	0.11	0.38	0.17	60
LM1913	0.65	0.61	0.28	0.49	0.33	−0.93	−1.17	0.15	0.18	0.16	61
LM21136	0.42	0.45	0.48	0.30	0.46	−1.22	−0.09	0.08	0.44	0.15	62
LM12	0.48	0.41	0.08	0.32	0.65	−1.26	−0.22	0.07	0.41	0.14	63
LM2123	0.43	0.49	0.20	0.47	0.27	−1.38	−0.61	0.04	0.32	0.10	64
YN2	0.54	0.65	0.26	0.32	0.21	−1.23	−1.40	0.08	0.12	0.09	65
LM9	0.51	0.58	0.19	0.31	0.28	−1.32	−1.14	0.05	0.19	0.08	66
YH6	0.51	0.49	0.08	0.28	0.38	−1.41	−0.92	0.03	0.24	0.07	67
LM11	0.53	0.61	0.04	0.42	0.15	−1.39	−1.50	0.03	0.10	0.05	68
HM29	0.49	0.56	0.06	0.22	0.25	−1.52	−1.32	0.00	0.14	0.03	69
LM21105	0.54	0.61	0.09	0.15	0.18	−1.52	−1.69	0.00	0.05	0.01	70

RWA, relative water absorption; WAR, seed water absorption rate; DR, seed dehiscence rate; GR, seed germination rate; GI, seed germination index. ^#^ The average of the salt tolerance index for each character under two NaCl treatments. * The weighted membership function value (D_1_ value).

**Table 3:** Salt tolerance index of each parameter and the comprehensive salt tolerance index of 35 wheat varieties at the germination and seedling stages under salt stress.

Variety (Line)	RWA ^#^	WAR	DR	GR	GI	PH	RL	SD	BM	Principal Component Value					Membership Function Value					Comprehensive Salt Tolerance Index * (D_2_ Value)
										X_1_	X_2_	X_3_	X_4_	X_5_	U_1_	U_2_	U_3_	U_4_	U_5_	
JM262	0.72	0.90	1.38	1.46	1.35	0.66	0.55	0.98	1.04	0.78	−0.25	2.57	0.55	−1.19	0.76	0.36	1.00	0.72	0.16	0.65
SD29	1.40	1.35	1.38	1.00	1.18	0.77	0.53	0.81	1.10	1.26	−1.87	−0.26	0.86	0.00	0.89	0.00	0.40	0.79	0.44	0.60
XM44	1.61	1.25	1.31	1.04	1.12	0.81	0.59	1.07	1.13	1.57	−1.57	−0.76	0.36	−0.36	0.97	0.07	0.29	0.67	0.35	0.61
JM60	0.73	0.77	1.50	1.25	1.29	0.45	0.55	0.99	1.17	0.51	−0.14	2.09	1.14	−0.56	0.68	0.38	0.90	0.86	0.31	0.64
ZM1860	1.35	1.32	1.22	1.17	0.93	0.60	0.86	0.84	1.19	1.16	−1.74	−0.08	−0.87	1.47	0.86	0.03	0.44	0.38	0.77	0.58
YN301	1.25	1.17	1.29	0.80	1.16	1.01	0.72	1.18	1.47	1.67	0.04	−1.18	0.47	0.09	1.00	0.42	0.20	0.70	0.46	0.69
JM54	1.22	1.16	1.50	0.94	0.88	0.46	0.41	0.89	1.33	0.55	−1.77	−0.49	1.35	0.74	0.69	0.02	0.35	0.91	0.61	0.54
YN999	0.92	0.89	1.19	1.05	1.11	0.76	0.64	1.07	1.01	0.60	0.10	0.26	0.12	−0.39	0.71	0.44	0.51	0.61	0.35	0.58
Emam	0.93	0.73	1.09	1.24	1.07	1.27	0.62	1.00	1.41	0.99	1.00	0.90	−0.27	−0.36	0.82	0.63	0.65	0.52	0.35	0.67
STM1	0.80	0.67	1.27	1.00	1.14	0.55	0.83	0.92	1.56	0.55	0.66	1.06	0.04	1.08	0.69	0.56	0.68	0.59	0.68	0.65
YM39	0.89	0.86	1.00	0.85	1.33	0.64	0.66	1.29	0.84	0.61	0.56	−0.36	−0.11	−1.58	0.71	0.54	0.38	0.56	0.07	0.54
LM30	0.82	0.69	1.03	0.94	1.25	0.74	0.71	0.88	1.18	0.35	0.68	0.71	−0.17	−0.12	0.64	0.56	0.61	0.54	0.41	0.58
YN1212	0.95	1.14	1.08	0.96	0.98	0.67	0.64	0.78	1.00	0.30	−0.89	−0.22	−0.27	0.61	0.62	0.22	0.41	0.52	0.58	0.50
SN41	0.47	0.52	1.50	1.03	1.01	1.49	0.56	1.18	1.34	0.70	2.66	0.66	1.59	−0.21	0.74	1.00	0.59	0.96	0.39	0.75
XM35	1.04	0.97	0.83	1.09	1.02	0.65	0.44	1.06	1.19	0.37	−0.63	−0.14	−0.75	−1.07	0.64	0.27	0.42	0.40	0.19	0.46
YM23	0.76	0.75	1.17	0.81	1.12	0.46	0.32	1.04	0.99	−0.26	−0.07	−0.10	1.15	−1.11	0.47	0.40	0.43	0.86	0.18	0.47
HM20	0.80	0.93	1.20	0.76	1.04	0.50	0.94	0.76	1.86	0.61	0.28	0.08	−0.28	2.46	0.71	0.47	0.47	0.52	1.00	0.64
ZM136	1.01	0.86	1.03	0.98	0.93	0.74	0.63	0.99	1.35	0.45	−0.01	−0.26	−0.42	0.37	0.67	0.41	0.40	0.48	0.52	0.54
HM168	0.75	0.74	1.09	1.01	1.00	0.63	1.02	1.04	1.02	0.35	0.90	0.35	−1.04	1.16	0.64	0.61	0.53	0.33	0.70	0.59
YM33	0.94	0.87	0.91	0.75	1.19	0.61	0.57	0.98	0.77	0.04	−0.06	−0.66	−0.10	−0.86	0.55	0.40	0.31	0.56	0.24	0.46
ZM15	0.80	0.93	0.77	1.01	1.02	0.39	0.43	1.03	0.91	−0.30	−0.65	−0.09	−0.78	−1.15	0.46	0.27	0.44	0.40	0.17	0.38
YJM586	0.74	0.72	0.75	0.96	1.16	0.62	0.51	0.87	0.81	−0.36	0.17	0.40	−0.66	−1.07	0.44	0.45	0.54	0.43	0.19	0.43
HM35	0.64	0.76	1.10	0.91	0.97	0.81	0.91	0.94	1.55	0.46	1.26	0.24	−0.61	1.56	0.67	0.69	0.51	0.44	0.79	0.64
NM21	0.79	0.81	0.48	1.13	0.95	0.58	0.72	0.69	1.31	−0.24	−0.15	0.69	−2.44	0.50	0.48	0.38	0.60	0.00	0.55	0.42
JM52	0.55	0.68	1.22	0.82	0.82	0.29	0.16	0.48	0.08	−1.98	−1.18	0.27	1.63	−0.10	0.00	0.15	0.51	0.97	0.41	0.27
RH520	0.73	0.94	0.65	0.94	0.94	0.85	0.55	1.22	0.66	−0.08	0.48	−1.03	−1.27	−1.38	0.52	0.52	0.24	0.28	0.12	0.41
WM206	0.63	0.65	1.22	0.60	0.91	0.53	0.21	1.08	1.22	−0.62	0.49	−0.97	1.74	−0.58	0.37	0.52	0.25	1.00	0.30	0.45
ZYGK1	0.41	0.34	0.94	0.94	0.99	0.37	0.41	0.52	0.28	−1.88	0.23	1.33	0.30	−0.21	0.03	0.46	0.74	0.66	0.39	0.32
LM1936	0.70	0.81	1.11	0.37	0.94	0.85	0.79	1.01	0.59	−0.28	1.23	−2.14	0.48	0.85	0.47	0.68	0.00	0.70	0.63	0.49
WY16	0.50	0.60	0.99	0.76	0.85	0.38	0.45	0.37	0.11	−1.97	−0.49	0.28	0.44	0.75	0.00	0.30	0.51	0.69	0.61	0.28
YM16	0.68	0.76	1.17	0.72	0.57	0.61	0.55	0.96	0.78	−0.83	0.23	−1.36	0.40	1.22	0.32	0.46	0.17	0.68	0.72	0.41
LM10	0.53	0.34	1.05	0.61	0.93	0.64	0.70	0.98	0.79	−0.86	1.78	−0.52	0.30	0.56	0.31	0.81	0.34	0.66	0.56	0.48
HY66	0.52	0.61	0.58	1.29	0.63	0.28	0.47	0.50	0.38	−1.93	−0.92	1.28	−1.87	0.40	0.02	0.21	0.73	0.14	0.53	0.22
TM178	0.67	0.66	0.98	0.62	0.80	0.40	0.32	0.49	0.22	−1.84	−0.72	−0.69	0.78	0.38	0.04	0.26	0.31	0.77	0.52	0.26
YM34	0.87	0.82	0.39	0.76	0.94	0.61	0.51	1.32	0.60	−0.44	0.38	−1.86	−1.79	−1.89	0.42	0.50	0.06	0.16	0.00	0.31

RWA, relative water absorption; WAR, seed water absorption rate; DR, seed dehiscence rate; GR, seed germination rate; GI, seed germination index; PH, plant height; RL, root length; SD, stem diameter; BM, biomass. ^#^ The average of the salt tolerance index for each character under two NaCl treatments. * The weighted membership function value (D_2_ value).

**Table 4:** Principal component feature vectors and contribution rates of the salt tolerance index for each morphological trait under salt stress.

Factor	Principal Component				
	PC1	PC2	PC3	PC4	PC5
RWA	0.73	−0.57	−0.28	−0.05	−0.02
WAR	0.67	−0.64	−0.28	−0.08	0.02
DR	0.44	−0.04	0.19	0.80	0.29
GR	0.35	−0.26	0.76	−0.29	−0.16
GI	0.62	0.10	0.34	0.13	−0.42
PH	0.61	0.52	−0.12	0.07	−0.07
RL	0.53	0.34	0.03	−0.43	0.54
SD	0.63	0.40	−0.34	−0.04	−0.45
BM	0.81	0.18	0.13	−0.04	0.27
Eigenvalue	3.39	1.40	1.04	0.95	0.86
Contribution rate/%	37.61	15.51	11.53	10.57	9.61
Accumulative contribution rate/%	37.61	53.12	64.65	75.22	84.83

RWA, relative water absorption; WAR, seed water absorption rate; DR, seed dehiscence rate; GR, seed germination rate; GI, seed germination index; PH, plant height; RL, root length; SD, stem diameter; BM, biomass.

**Table 5:** Name of the test wheat varieties.

Serial Number	Name	Breeding Region	Serial Number	Name	Breeding Region
1	Huaimai 29/HM29	Jiangsu	36	Xumai 35/XM35	Jiangsu
2	Ningmai 21/NM21	Jiangsu	37	Ruihuamai 502/RHM502	Jiangsu
3	Yangfumai 4/YFM4	Jiangsu	38	Huamai 2892/HM2892	Jiangsu
4	Lianmai 1905/LM1905	Jiangsu	39	Huamai 17/HM17	Jiangsu
5	Lianmai 1825/LM1825	Jiangsu	40	Huamai 16/HM16	Jiangsu
6	Lianmai 9/LM9	Jiangsu	41	Huamai 29262/HM29262	Jiangsu
7	Lianmai 2193/LM2193	Jiangsu	42	Wanmai 206/WM206	Anhui
8	Lianmai 12/LM12	Jiangsu	43	Huiyan 66/HY66	Anhui
9	Lianmai 231/LM231	Jiangsu	44	Huiyan 1722/HY1722	Anhui
10	Lianmai 21136/LM21136	Jiangsu	45	Womai 203/WM203	Anhui
11	Lianmai 1901/LM1901	Jiangsu	46	Woyu 16/WY16	Anhui
12	Lianmai 186/LM186	Jiangsu	47	Xinshiji 999/XSJ999	Anhui
13	Lianmai 21105/LM21105	Jiangsu	48	Zhongyuanguoke 1/ZYGK1	Anhui
14	Lianmai 2123/LM2123	Jiangsu	49	Lemai G1302/LMG1302	Anhui
15	Lianmai 10/LM10	Jiangsu	50	Yanghong 6/YH6	Anhui
16	Lianmai 11/LM11	Jiangsu	51	Jimai 52/JM52	Shandong
17	Lianmai 21137/LM21137	Jiangsu	52	Jimai 54/JM54	Shandong
18	Lianmai 1913/LM1913	Jiangsu	53	Jimai 60/JM60	Shandong
19	Lianmai 1738/LM1738	Jiangsu	54	Jimai 262/JM262	Shandong
20	Lianmai 1936/LM1936	Jiangsu	55	Jimai 379/JM379	Shandong
21	Yangmai 25/YM25	Jiangsu	56	Yannong 301/YN301	Shandong
22	Yangjiangmai 586/YJM586	Jiangsu	57	Yannong 1212/YN1212	Shandong
23	Yangmai 34/YM34	Jiangsu	58	Yannong 999/YN999	Shandong
24	Yangmai 16/YM16	Jiangsu	59	Shannong 41/SN41	Shandong
25	Yangmai 39/YM39	Jiangsu	60	Zhongxinmai 998/ZXM998	Hebei
26	Yangmai 33/YM33	Jiangsu	61	Tianmai 178/TM178	Henan
27	Yangnuomai 2/YNM2	Jiangsu	62	Suimai 132/SM132	Henan
28	Ruihua 520/RH520	Jiangsu	63	Shangdao 29/SD29	Henan
29	Zhengmai 15/ZM15	Jiangsu	64	Zhengmai 136/ZM136	Henan
30	Yangmai 23/YM23	Jiangsu	65	Zhengmai 1860/ZM1860	Henan
31	Sutaimai 1/STM1	Jiangsu	66	Ronghua 116/RH116	Shaanxi
32	Xumai 44/XM44	Jiangsu	67	Weilong 169/WL169	Shaanxi
33	Huaimai 35/HM35	Jiangsu	68	Longmai 30/LM30	Heilongjiang
34	Huaimai 20/HM20	Jiangsu	69	Elnilein	Northern Sudan
35	Huaimai 168/HM168	Jiangsu	70	Emam	Northern Sudan

## Data Availability

Data are contained within the article and Supplementary Materials.

## Supplementary Materials

The following supporting information can be downloaded at: https://www.mdpi.com/article/10.3390/plants13223201/s1, Table S1: Salt tolerance index of each morphological parameter in 70 wheat varieties at the germination stage under two salt treatments; Table S2: Salt tolerance index of each morphological parameter in 35 wheat varieties at the seedling stage under two salt treatments; Table S3: The main agronomic characteristics of wheat varieties.

## Abbreviations

BM	Biomass
CK	Control
DR	Seed dehiscence rate
GI	Seed germination index
GR	Seed germination rate
PC	Principal component
PCA	Principal component analysis
PH	Plant height
RL	Root length
RWA	Relative water absorption
SD	Stem diameter
WAR	Seed water absorption rate

## References

[1] Li W.B., Wang D.Y., Li H., Liu S.H. (2017). Urbanization-induced site condition changes of peri-urban cultivated land in the black soil region of northeast China. Ecol. Indic..

[2] Chao Z.H., Zhang P.D., Wang X.F. (2018). Impacts of urbanization on the net primary productivity and cultivated land change in Shandong Province, China. J. Indian Soc. Remote Sens..

[3] Yang S.F., Hu S.G., Li W.D., Zhang C.R., Song D.D. (2020). Spatio-temporal nonstationary effects of impact factors on industrial land price in industrializing cities of China. Sustainability.

[4] Wang Y.F. (2022). Effects of urbanization on spatial-temporal changes of cultivated land in Bohai Rim region. Environ. Dev. Sustain..

[5] Zhang J.Z., He C.X., Chen L., Cao S.X. (2018). Improving food security in China by taking advantage of marginal and degraded lands. J. Clean. Prod..

[6] Xu Z.C., Ren T.T., Marowa P., You X.W., Lu X.L., Li Y.Q., Zhang C.S. (2020). Establishment of a cultivation mode of glycine soja, the bridge of phytoremediation and industrial utilization. Agronomy.

[7] Xu W., Dong Y.E., Teng X., Zhang P.P. (2018). Evaluation of the development intensity of China’s coastal area. Ocean Coast. Manag..

[8] Donnelly A., Rodríguez-Rodríguez D. (2022). Effectiveness of protected areas against land development in coastal areas of the Mediterranean global biodiversity hotspot. Glob. Ecol. Conserv..

[9] Chen Y.S., Sun Z.F., Wang Y.M., Ma Y., Zhou Y.W. (2024). The green development in saline–alkali lands: The evolutionary game framework of small farmers, family farms, and seed industry enterprises. Land.

[10] Zhang J.S., Jiang X.L., Miao Q., Yu B.T., Xu L.M., Cui Z.L. (2019). Combining mineral amendments improves wheat yield and soil properties in a coastal saline area. Agronomy.

[11] Wang N., Zang J.L., Guo X.R., Wang H.Y., Huang N.J., Zhao C.K., Zhao X.Q., Liu J.P. (2022). Role of rice cultivation on fluorine distribution behavior in soda saline-alkali land. Sci. Total Environ..

[12] Khare T., Jamla M., Mathur V., Kumar V. (2024). Exploring halobiome resources for developing salt-tolerant crops: A perspective review. J. Plant Growth Regul..

[13] Islam M.R., Sarker M.R.A., Sharma N., Rahman M.A., Collard B.C.Y., Gregorio G.B., Ismail A.M. (2016). Assessment of adaptability of recently released salt tolerant rice varieties in coastal regions of South Bangladesh. Field Crops Res..

[14] Niu G.H., Rodriguez D.S., Cabrera R., Jifon J., Leskovar D., Crosby K. (2010). Salinity and soil type effects on emergence and growth of pepper seedlings. HortScience.

[15] Feghhenabi F., Hadi H., Khodaverdiloo H., Genuchten M.T.V. (2020). Seed priming alleviated salinity stress during germination and emergence of wheat (*Triticum aestivum* L.). Agr. Water Manag..

[16] Xie H.Y., Li J., Zhang Y.T., Xu X.B., Wang L.Q., Ouyang Z. (2021). Evaluation of coastal farming under salinization and optimized fertilization strategies in China. Sci. Total Environ..

[17] Wang H., Zheng C.L., Ning S.R., Cao C.Y., Li K.J., Dang H.K., Wu Y.Q., Zhang J.P. (2023). Impacts of long-term saline water irrigation on soil properties and crop yields under maize-wheat crop rotation. Agr. Water Manag..

[18] Long S.P., Marshall-Colon A., Zhu X.G. (2015). Meeting the global food demand of the future by engineering crop photosynthesis and yield potential. Cell.

[19] Zhao C.Z., Zhang H., Song C.P., Zhu J.K., Shabala S. (2020). Mechanisms of plant responses and adaptation to soil salinity. Innovation.

[20] Alkharabsheh H.M., Seleiman M.F., Hewedy O.A., Battaglia M.L., Jalal R.S., Alhammad B.A., Schillaci C., Ali N., Al-Doss A. (2021). Field crop responses and management strategies to mitigate soil salinity in modern agriculture: A review. Agronomy.

[21] Hmissi M., Chaieb M., Krouma A. (2023). Differences in the physiological indicators of seed germination and seedling establishment of durum wheat (*Triticum durum* Desf.) cultivars subjected to salinity stress. Agronomy.

[22] Choudhary A., Kaur N., Sharma A., Kumar A. (2021). Evaluation and screening of elite wheat germplasm for salinity stress at the seedling phase. Physiol. Plant..

[23] Hasanuzzaman M., Saha N.R., Farabi S., Tahjib-Ul-Arif M., Yasmin S., Haque M.S. (2023). Screening of salt-tolerant wheat (*Triticum aestivum* L.) through morphological and molecular markers. Cereal Res. Commun..

[24] Ru S.S., Bernardo R. (2019). Targeted recombination to increase genetic gain in self-pollinated species. Theor. Appl. Genet..

[25] Moustafa E.S.A., Ali M.M.A., Kamara M.M., Awad M.F., Hassanin A.A., Mansour E. (2021). Field screening of wheat advanced lines for salinity tolerance. Agronomy.

[26] Horak M.J., Sweat J.K. (1994). Germination, emergence, and seedling establishment of Buffalo Gourd (*Cucurbita foetidissima*). Weed Sci..

[27] Gardarin A., Coste F., Wagner M.H., Dürr C. (2016). How do seed and seedling traits influence germination and emergence parameters in crop species? A comparative analysis. Seed Sci. Res..

[28] Soleymani A. (2019). Safflower (*Carthamus tinctorius* L.) seed vigor tests for the prediction of field emergence. Ind. Crops Prod..

[29] Kiremi M.S., Arslan H., Sezer İ., Akay H. (2022). Evaluating and modeling of the seedling growth ability of wheat seeds as affected by shallow-saline groundwater conditions. Gesunde Pflanz..

[30] Nakhla W.R., Sun W.Q., Fan K., Yang K., Zhang C.P., Yu S.B. (2021). Identification of QTLs for salt tolerance at the germination and seedling stages in rice. Plants.

[31] Vuković R., Čamagajevac I.Š., Vuković A., Šunić K., Begović L., Mlinarić S., Sekulić R., Sabo N., Španić V. (2022). Physiological, biochemical and molecular response of different winter wheat varieties under drought stress at germination and seedling growth stage. Antioxidants.

[32] Li L., Peng Z., Mao X.G., Wang J.Y., Li C.N., Chang X.P., Jing R.L. (2021). Genetic insights into natural variation underlying salt tolerance in wheat. J. Exp. Bot..

[33] Zaari Jabri N., Ait-El-Mokhtar M., Mekkaoui F., Amghar I., Achemrk O., Diria G., Hmyene A. (2023). Impacts of cadmium toxicity on seed germination and seedling growth of *Triticum durum* cultivars. Cereal Res. Commun..

[34] Abdel Latef A.A.H., Abu Alhmad M.F., Kordrostami M., Abo-Baker A.B., Zakir A. (2020). Inoculation with *Azospirillum lipoferum* or *Azotobacter chroococcum* reinforces maize growth by improving physiological activities under saline conditions. J. Plant Growth Regul..

[35] Zhang Z.C., Xia Z.L., Zhou C.J., Wang G., Meng X., Yin P.C. (2024). Insights into salinity tolerance in wheat. Genes.

[36] Gao S.A., Tian R.M., Jia H.N., Wang M., Liu D.Z., Lu Q.M., Lu S., Gao Z.P., Wan F.Q., Zhang X.B. (2024). Identification of salt tolerance of 236 wheat germplasm at seedling stage and screening of salt tolerance indicators. J. Triticeae Crops.

[37] Gupta C., Salgotra R.K. (2022). Epigenetics and its role in effecting agronomical traits. Front. Plant Sci..

[38] Zhang D.L., Gan Y.J., Le L., Pu L. Epigenetic variation in maize agronomical traits for breeding and trait improvement. J. Genet. Genom..

[39] Zhao S.S., Zhang Q.K., Liu M.Y., Zhou H.P., Ma C.L., Wang P.P. (2021). Regulation of plant responses to salt stress. Int. J. Mol. Sci..

[40] Khalid M.F., Huda S., Yong M., Li L.H., Chen Z.H., Ahmed T. (2023). Alleviation of drought and salt stress in vegetables: Crop responses and mitigation strategies. Plant Growth Regul..

[41] Ibrahim M.E.H., Zhu X.K., Zhou G.S., Nimir N.E.A. (2016). Comparison of germination and seedling characteristics of wheat varieties from China and Sudan under salt stress. Agron. J..

[42] Hou L.J., Liu Z.H., Zhang D.Z., Liu S.H., Chen Z.Z., Wu Q.F., Shang Z.Z., Wang J.S., Wang J.W. (2024). BR regulates wheat root salt tolerance by maintaining ROS homeostasis. Planta.

[43] Munns R., James R.A. (2003). Screening methods for salinity tolerance: A case study with tetraploid wheat. Plant Soil..

[44] Xu Y.M., Bu W.C., Xu Y.C., Fei H., Zhu Y.M., Ahmad I., Nimir N.E.A., Zhou G.S., Zhu G.L. (2024). Effects of salt stress on physiological and agronomic traits of rice genotypes with contrasting salt tolerance. Plants.

[45] Sun J.Y., Cheng Q., Li S.M. (2017). Effect of salt stress on seed germination and seedling growth of wheat. Mol. Plant Breed..

[46] Hu H.R., Liu H., Liu F.H. (2018). Seed germination of hemp (*Cannabis sativa* L.) cultivars responds differently to the stress of salt type and concentration. Ind. Crop. Prod..

[47] Zhang H.H., Xu N., Wu X.Y., Wang J.R., Ma S.L., Li X., Sun G.Y. (2018). Effects of four types of sodium salt stress on plant growth and photosynthetic apparatus in sorghum leaves. J. Plant Interact..

[48] Flowers T. (2004). Improving crop salt tolerance. J. Exp. Bot..

[49] Abulela H.A., El Shafee E., Farag H.M., Yacoub I.H., Elarabi N.I. (2022). Evaluation of the morpho-physiological traits and the genetic diversity of some Egyptian bread wheat cultivars under salt stress conditions. Cereal Res. Commun..

[50] Wu L.M., Du J.G., Zhang Y.D., Xue Y.Q., Jiang C.Y., Lu W., Zheng Y.X., Zhou C.B., Xiong A.S., Li M.Y. (2024). Identification and evaluation of celery germplasm resources for salt tolerance. Agronomy.

[51] Peng Z., Li P., Liu Y.P., Liu H.M., Jing R.L. (2017). Evaluation of salinity tolerance in wheat (*Triticum aestium*) genotypes at germination and seedling stages. J. Plant Genet. Resour..

[52] Karami E., Sartip A., Arshadi A., Zare M. (2021). Comparing the potential of indices and multivariate statistical techniques to select drought tolerant genotypes in barley (*Hordeum vulgare* L.). Braz. J. Bot..

[53] Shamuyarira K.W., Shimelis H., Figlan S., Chaplot V. (2022). Path coefficient and principal component analyses for biomass allocation, drought tolerance and carbon sequestration potential in wheat. Plants.

[54] Pan Y.L., Li Y.F., Liu Z.Y., Zou J.T., Li Q. (2022). Computational genomics insights into cold acclimation in wheat. Front. Genet..

[55] Tahjib-Ul-Arif M., Sayed M.A., Islam M.M., Siddiqui M.N., Begum S.N., Hossain M.A. (2018). Screening of rice landraces (*Oryza sativa* L.) for seedling stage salinity tolerance using morpho-physiological and molecular markers. Acta. Physiol. Plant.

[56] Tian H.J., Liu H., Zhang D., Hu M.T., Zhang F.L., Ding S.Q., Yang K.Z. (2024). Screening of salt tolerance of maize (*Zea mays* L.) lines using membership function value and GGE biplot analysis. PeerJ.

[57] Sivakumar J., Prashanth J.E.P., Rajesh N., Reddy S.M., Pinjari O.B. (2020). Principal component analysis approach for comprehensive screening of salt stress-tolerant tomato germplasm at the seedling stage. J. Biosci..

[58] Li Y.Y., Chen B., Yao L.R., Di X.T., Si E.J., Wang J.C., Ma X.L., Meng Y.X., Wang H.J., Li B.C. (2021). Evaluation of salt and alkali tolerance and germplasm screening of 283 wheat varieties (lines) during germination. J. Agric. Sci. Technol..

[59] Han R., Xie S.B., Li X., Guo X.F., Gong W.P., Wang X.L., Liu A.F., Li H.S., Liu C., Liu J.J. (2020). Screening, identification and evaluation of salt-tolerant wheat germplasms. Shandong Agric. Sci..

[60] Quan X.Y., Liang X.L., Li H.M., Xie C.J., He W.X., Qin Y.X. (2021). Identification and characterization of wheat germplasm for salt tolerance. Plants.

[61] Tao R.R., Ding J.F., Li C.Y., Zhu X.K., Guo W.S., Zhu M. (2021). Evaluating and screening of agro-physiological indices for salinity stress tolerance in wheat at the seedling stage. Front. Plant Sci..

[62] El-Hendawy S.E., Ruan Y., Hu Y., Schmidhalter U. (2009). A comparison of screening criteria for salt tolerance in wheat under field and controlled environmental conditions. J. Agron. Crop Sci..

[63] El-Hendawy S.E., Hassan W.M., Al-Suhaibani N.A., Refay Y., Abdella K.A. (2017). Comparative performance of multivariable agro-physiological parameters for detecting salt tolerance of wheat cultivars under simulated saline field growing conditions. Front. Plant Sci..

[64] Ti J.S., Tong W.J., Wang Y.H., Zhou Y.Y., Wen X.Y., Chen F. (2013). Screening and evaluation of salinity tolerance index for wheat in Hetao Irrigation District. J. China Agric. Univ..

[65] Griffa S., Ribotta A., López Colomba E., Tommasino E., Carloni E., Luna C., Grunberg K. (2010). Evaluation seedling biomass and its components as selection criteria for improving salt tolerance in Buffel grass genotypes. Grass Forage Sci..

[66] Ma Y.C., Li B.W., Yang D.J., Wang S.G., Yu L.X., Zhan H., Li J. (2023). An optimal genomic DNA extraction method for shoots of four Dendrocalamus species based on membership function analysis. Biotechniques.

