# Supplementary material for: Screening and Evaluation of Salt-Tolerant Wheat Germplasm Based on the Main Morphological Indices at the Germination and Seedling Stages
Source: Plants (Basel). 2024 Nov 14;13(22):3201. doi: 10.3390/plants13223201 (PMC11598134; doi:10.3390/plants13223201)
Supplement: Supplementary file 1 [file plants-13-03201-s001.zip › plants-3230884-supplementary.pdf]

Supplementary Tables

Table S1. Salt tolerance index of each morphological parameter in 70 wheat varieties at the germination stage under two salt treatments.

Variety	140 mM NaCl					180 mM NaCl				
	Relative water absorption	Seed water absorption rate	Seed dehiscence rate	Seed germination rate	Seed germination index	Relative water absorption	Seed water absorption rate	Seed dehiscence rate	Seed germination rate	Seed germination index
HM29	0.53	0.61	0.06	0.41	1.19	0.46	0.52	0.06	0.03	0.21
NM21	0.93	0.96	0.63	1.29	1.11	0.65	0.66	0.33	0.97	0.84
YFM4	0.93	0.93	0.60	0.76	1.31	0.64	0.63	0.68	0.29	0.80
LM1905	0.78	0.60	0.54	0.83	1.31	0.29	0.30	0.09	0.00	0.29
LM1825	0.50	0.57	0.71	0.60	0.98	0.49	0.56	0.09	0.00	0.42
LM9	0.73	0.83	0.27	0.63	1.10	0.29	0.33	0.11	0.00	0.15
LM2193	0.79	0.83	0.76	0.56	0.96	0.56	0.38	0.32	0.56	0.38
LM12	0.59	0.50	0.17	0.64	1.03	0.37	0.32	0.00	0.00	0.55
LM231	0.79	0.82	0.35	0.34	1.12	0.47	0.49	0.06	0.01	0.58
LM21136	0.48	0.52	0.56	0.59	1.12	0.36	0.38	0.40	0.00	0.40
LM1901	0.67	0.74	1.08	0.52	1.29	0.63	0.70	0.63	0.14	0.64
LM186	1.06	1.03	1.13	0.38	1.20	0.85	0.83	0.66	0.03	0.76
LM21105	0.62	0.70	0.15	0.30	1.20	0.47	0.52	0.02	0.00	0.12
LM2123	0.56	0.63	0.39	0.51	1.08	0.31	0.35	0.01	0.43	0.16
LM10	0.61	0.17	1.07	0.63	0.93	0.45	0.52	1.03	0.59	0.85
LM11	0.58	0.66	0.08	0.51	0.88	0.49	0.56	0.01	0.33	0.12
LM21137	0.66	0.70	0.44	0.60	1.06	0.56	0.59	0.42	0.48	0.75
LM1913	0.67	0.64	0.41	0.89	1.21	0.62	0.59	0.15	0.09	0.25
LM1738	0.49	0.48	0.46	1.03	0.97	0.50	0.50	0.36	0.14	0.95
LM1936	0.64	0.75	1.03	0.49	1.02	0.75	0.87	1.19	0.24	0.72
YM25	0.73	0.63	0.43	0.53	1.32	0.77	0.65	0.40	0.62	1.00

YJM586	0.70	0.69	1.04	1.10	0.94	0.78	0.76	0.47	0.83	1.00
YM34	0.91	0.85	0.37	0.96	0.96	0.84	0.78	0.41	0.57	1.00
YM16	0.72	0.80	1.13	0.87	1.06	0.64	0.71	1.20	0.58	0.58
YM39	0.91	0.88	0.96	0.72	0.83	0.88	0.85	1.04	0.99	1.36
YM33	0.92	0.86	0.89	0.89	0.90	0.96	0.89	0.93	0.62	1.18
YN2	0.60	0.71	0.32	0.35	0.91	0.49	0.58	0.19	0.29	0.17
RH520	0.69	0.89	0.63	0.81	1.01	0.77	0.99	0.67	1.06	0.98
ZM15	0.80	0.94	0.58	1.28	1.01	0.79	0.92	0.97	0.75	1.10
YM23	0.78	0.77	1.37	0.95	1.16	0.75	0.73	0.97	0.66	1.18
STM1	0.99	0.84	1.23	1.02	0.82	0.60	0.50	1.31	0.97	1.16
XM44	1.45	1.13	1.25	1.00	0.88	1.76	1.37	1.38	1.08	1.05
HM35	0.63	0.74	1.05	0.81	0.87	0.66	0.77	1.16	1.01	0.98
HM20	0.81	0.95	1.12	0.79	0.56	0.78	0.91	1.28	0.73	1.05
HM168	0.86	0.85	1.11	1.07	0.76	0.64	0.63	1.07	0.95	1.04
XM35	0.94	0.88	0.67	1.18	0.93	1.14	1.06	0.98	1.01	0.85
RH502	0.52	0.48	0.41	0.64	0.68	0.17	0.16	0.49	0.92	0.82
HM2892	0.39	0.33	0.71	0.24	0.75	0.28	0.24	0.29	0.26	0.78
HM17	0.34	0.27	1.04	0.89	1.23	0.16	0.13	0.61	0.19	0.67
HM16	0.78	0.75	0.70	0.35	0.71	0.37	0.36	0.38	0.28	0.55
HM29262	0.34	0.35	0.77	0.11	0.82	0.57	0.59	0.53	0.04	0.52
WM206	0.68	0.70	1.13	0.85	0.96	0.59	0.60	1.30	0.36	0.92
HY66	0.68	0.81	0.76	1.14	0.89	0.35	0.41	0.39	1.43	0.49
HY1722	0.68	0.79	0.79	0.67	0.82	0.31	0.36	0.20	0.32	0.49
WM203	0.87	0.90	0.35	0.66	1.08	0.60	0.62	0.38	0.54	0.82

WY16	0.51	0.61	1.14	0.79	0.83	0.49	0.59	0.84	0.73	0.89
XSJ999	0.41	0.46	0.61	1.02	0.67	0.22	0.24	0.54	0.95	0.62
ZYGK1	0.60	0.51	0.96	0.94	0.75	0.21	0.18	0.92	0.95	0.97
LMG1302	0.48	0.41	1.10	0.67	0.73	0.34	0.29	1.16	0.32	0.44
YH6	0.55	0.54	0.14	0.51	0.62	0.46	0.44	0.02	0.06	0.27
JM52	0.52	0.63	1.16	0.79	0.77	0.59	0.72	1.28	0.85	0.81
JM54	1.24	1.19	1.39	1.40	0.86	1.20	1.14	1.60	0.48	0.80
JM60	0.69	0.73	1.37	1.25	0.87	0.77	0.81	1.63	1.25	1.28
JM262	0.75	0.94	1.18	1.21	0.73	0.70	0.87	1.58	1.71	1.38
JM379	0.48	0.51	1.32	0.40	0.86	0.47	0.49	1.03	0.32	0.64
YN301	1.37	1.28	1.20	0.89	0.62	1.13	1.05	1.38	0.71	1.22
YN1212	1.31	1.57	1.19	0.98	0.82	0.59	0.70	0.98	0.95	0.87
YN999	1.03	1.00	1.18	0.98	0.85	0.82	0.79	1.20	1.12	1.10
SN41	0.45	0.50	1.42	0.96	0.69	0.49	0.55	1.58	1.10	1.08
ZX998	0.83	0.78	0.84	0.61	0.78	0.41	0.38	0.82	0.61	0.60
TM178	0.98	0.96	1.07	0.62	0.42	0.36	0.35	0.89	0.62	0.73
SM132	0.55	0.54	0.84	0.31	0.52	0.28	0.27	0.83	0.12	0.73
SD29	1.31	1.27	1.19	0.96	0.74	1.49	1.43	1.58	1.04	1.24
ZM136	0.93	0.79	0.96	0.92	0.38	1.09	0.93	1.10	1.04	0.97
ZM1860	1.40	1.38	1.23	1.08	0.25	1.29	1.26	1.21	1.26	0.88
RH116	0.55	0.57	0.67	0.30	0.41	0.30	0.30	0.54	0.12	0.62
WL169	0.84	0.83	1.10	0.35	0.49	0.49	0.48	0.96	0.20	0.49
LM30	0.78	0.66	1.07	1.09	0.18	0.87	0.73	0.99	0.79	1.30

Elnilein	0.73	0.64	0.78	0.31	0.28	0.38	0.33	0.67	0.16	0.41
LM21105	1.05	0.82	1.07	1.26	0.23	0.81	0.63	1.12	1.22	1.11

Salt tolerance index = (the value under the NaCl treatment/the value under the CK) × (the value under the NaCl treatment/the mean value of all germplasms under the NaCl treatment).

Table S2. Salt tolerance index of each morphological parameter in 35 wheat varieties at the seedling stage under two salt treatments.

Variety	140 mM NaCl				180 mM NaCl			
	Plant height	Root length	Stem diameter	Biomass	Plant height	Root length	Stem diameter	Biomass
JM262	0.77	0.55	0.91	1.19	0.55	0.56	1.05	0.89
SD29	0.85	0.67	0.83	1.14	0.69	0.39	0.79	1.05
XM44	1.17	0.76	1.03	1.15	0.45	0.42	1.11	1.11
JM60	0.49	0.49	0.97	1.39	0.40	0.61	1.02	0.95
ZM1860	0.58	1.27	0.78	1.32	0.61	0.44	0.89	1.06
YN301	0.93	0.61	1.08	1.69	1.08	0.82	1.29	1.25
JM54	0.61	0.35	0.88	1.58	0.31	0.48	0.90	1.08
YN999	0.82	0.87	0.96	1.08	0.69	0.41	1.17	0.94
Emam	1.27	0.65	0.96	1.47	1.27	0.58	1.03	1.36
STM1	0.44	1.36	0.88	1.83	0.66	0.29	0.96	1.28
YM39	0.55	0.66	1.14	1.00	0.74	0.66	1.44	0.67
LM30	0.82	0.67	0.89	1.27	0.66	0.75	0.87	1.09
YN1212	0.75	0.86	0.76	1.26	0.60	0.43	0.79	0.74
SN41	1.22	0.49	1.31	1.41	1.75	0.63	1.05	1.28
XM35	0.73	0.53	1.21	1.36	0.56	0.34	0.92	1.03
YM23	0.53	0.31	0.95	1.03	0.39	0.33	1.13	0.94
HM20	0.53	1.58	0.75	2.30	0.47	0.31	0.77	1.41
ZM136	0.64	0.89	0.92	1.30	0.84	0.38	1.06	1.39
HM168	0.57	1.74	1.41	1.13	0.69	0.30	0.67	0.91
YM33	0.57	0.71	0.97	0.89	0.65	0.43	0.99	0.65
ZM15	0.38	0.42	0.95	0.93	0.40	0.44	1.10	0.89
YJM586	0.61	0.53	0.78	1.01	0.63	0.50	0.96	0.62
HM35	1.11	1.41	0.91	1.82	0.51	0.41	0.97	1.28
NM21	0.62	0.77	0.64	1.37	0.55	0.67	0.74	1.24
JM52	0.57	0.31	0.89	0.15	0.00	0.02	0.06	0.00
RH520	0.85	0.60	1.11	0.78	0.86	0.49	1.32	0.55
WM206	0.60	0.23	1.03	1.36	0.46	0.19	1.13	1.09
ZYGK1	0.48	0.55	0.60	0.43	0.27	0.27	0.43	0.12
LM1936	1.01	0.83	0.92	0.60	0.69	0.75	1.10	0.59
WY16	0.52	0.55	0.41	0.18	0.24	0.36	0.33	0.04
YM16	0.68	0.41	0.87	0.94	0.53	0.68	1.05	0.61
LM10	0.63	0.86	0.89	0.88	0.64	0.53	1.08	0.70
HY66	0.31	0.55	0.65	0.53	0.25	0.38	0.34	0.24
TM178	0.61	0.40	0.52	0.38	0.19	0.23	0.45	0.06
YM34	0.52	0.52	1.26	0.72	0.70	0.49	1.38	0.48

Salt tolerance index = (the value under the NaCl treatment/the value under the CK) × (the value under the NaCl treatment/the mean value of all germplasms under the NaCl treatment).

Table S3. The main agronomic characteristics of wheat varieties.

Variety	Breeding unit	Source	Main agronomic characteristics
HM29	Huaiyin Institute of Agricultural Sciences of the Xuhuai District of Jiangsu Province, Huaian, China	Huaimai 20/Mianyang 04254	A semi-winter one with middle and late maturing. The seedlings were creeping, the tillering ability was strong, and the percentage of effective tillers was average. The plant height is about 90 cm, the plant type is semi-loose, the flag leaves are washed, the plant is waxy, the appearance is beautiful, the light transmission between the lines is good, the stalk elasticity is general. The ear layer is thick, the ear is multiple and even, the ear is small, and the fruit is good. Spindles, long awns, white shell, white grain, kernels horny, evenly full. The average number of ears per mu, the number of grains per ear is 32.9 and 1000-grain weight is 41.7 g.
YFM4	Agricultural Science Institute of Jiangsu Lixiahe District, Yangzhou, China	Ningmai 8/Ningmai 9	A spring wheat with mid-early maturity and growth period of 216 days. The seedlings were upright, with strong tillering and high percentage of effective tillers. The plant type is compact, the plant height is 87 cm, the stem is thick, and the lodging resistance is strong. Oblong ear, long awns, white shell, kernels pink, semi-horny. Effective panicle is 310,000 per mu, the number of grains per panicle is 41, and 1000-grain weight is 41 g.
LM1905 (Lianmai 15)	Lianyungang Academy of Agricultural Sciences, Lianyungang, China	Lian 0809//Lian 0318/Weimai 10	A semi-winter variety with the whole growth period of 222.2 days. The seedlings are semi-creeping, slender leaves, dark green leaves, and strong tillering ability. The plant height is 80.2 cm, the plant type is loose, lodging resistance is better, the uniformity is good, the ear layer is neat, and the ripening is good. Spindles, long awns, white grains, hard grains, medium fullness. The average number of ears per mu is 418,000, the number of grains per ear is 37.6, and the 1000-grain weight is 41.6 g.
LM1825 (Lianmai 185)	Lianyungang Academy of Agricultural Sciences, Lianyungang 222000, China	Lianmai 0927/Huaimai 0559//Lianmai 1011	A semi-winter variety with the whole growth period of 227.5 days. The seedlings are half creeping and have strong tillering ability. The plant height is 91.6 cm, the plant type is compact, the leaves are upthrust, the ear layer is orderly, and the ripening is relatively good. Spindle-shaped, waxy weight, long awns, white shells, white grains, keratin grains, the average acre effective ear 422,000, 34.5 grains per ear, 1000-grain weight 44.5 g.
LM9	Lianyungang Academy of Agricultural Sciences, Lianyungang 222000, China	Lian 0608/Lian 0709	A semi-winter one with the whole growth period of 222.0 days. The seedlings are semi-creeping, narrow leaves, dark green leaves and strong tillering ability. The plant height is 81.8 cm, the plant type is compact, lodging resistance is better. The uniformity is good, the ear layer is more orderly, and the ripening phase is general. Spindles, long awns, white shell, white grain, kernels half horny, good fullness. The number of ears in mu is 432,000, the number of grains per ear is 33.1, and the 1000-grain weight is 41.8 g.
LM12	Lianyungang Academy of Agricultural Sciences, Lianyungang 222000, China	Liuhu 98/Xinong 4711//Shengxuan 6/Luo 38	A semi-winter one with early maturing and the whole growth period of 227.3 days. The seedlings are semi-creeping and the leaves are green. Strong tillering ability and moderate cold resistance. The plant height is 88.6 cm, the plant type is compact, the ear layer is neat, lodging resistance is medium. Good-looking, get up earlier, better than familiar. Spindles, long awns, white shell, white grain, horny. The average effective ear of mu was 419,000, with 36.5 grains per ear and 1000-grain weight of 40.1 g.

LM186	Lianyungang Academy of Agricultural Sciences, Lianyungang 222000, China	Yan 0953/Xinong 538	A semi-winter one with the whole growth period of 227.5 days. The seedlings are half crawling and have strong tillering ability. The plant height is 91.6 cm, the plant type is compact, the leaves are upthrust, the ear layer is orderly, and the ripening is relatively good. Spicule shape, waxy weight, long awns, white shell, white grain, keratin. The average effective ear of mu was 422,000, with 34.5 grains per ear and 1000-grain weight of 44.5 g.
LM10	Lianyungang Academy of Agricultural Sciences, Lianyungang 222000, China	Lian 0608/Lian 0709	A semi-winter one with the whole growth period of 226.1 days. The seedlings are semi-creeping and the leaves are green. The tillering ability is strong and the cold resistance is good. The plant height is 87.8 cm, the plant type is compact, the ear layer is neat, lodging resistance is better. Grout fast, ripeness is better. Spindles, long awns, white shells, white grains, kernels horny. The average acre effective ear 407,000, with 36.5 grains per ear and 1000-grain weight of 40.1 g.
LM11	Lianyungang Academy of Agricultural Sciences, Lianyungang 222000, China	20005/Dong A4	A semi-winter one with the whole growth period of 229.6 days. The seedlings are half crawling and have strong tillering ability. The plant height was 90.0 cm, the plant type was loose, the panicle layer was neat, and the panicle number was more. Spindles, long awns, white shell, white grain, horny. The average effective ear of mu was 438,000, with 34.6 grains per ear and 1000-grain weight of 40.7 g.
LM1913 (Lianmai 13)	Lianyungang Academy of Agricultural Sciences, Lianyungang 222000, China	Lianmai 0927/Haimai 0559//Lianmai 1011	A semi-winter one with the whole growth period is 224.7 days. The seedlings are half crawling, and their strength is strong. The plant height is 81.9 cm, the plant type is compact, the ear layer is neat, and the ripening is better. Spindles, long awns, white shells, white grains, hard grains. The average effective ear of mu was 407,000, with 37.5 grains per ear and 1000-grain weight of 42.0 g.
YM25	Agricultural Science Institute of Jiangsu Lixiahe District, Yangzhou, China	Yang 17*2//Yang 11/Yumai 18	A spring wheat variety with a whole growth period of 202 days. The seedlings are half creeping, strong tillering and vigorous growth. The plant type is more compact, the leaves are raised, the ear layer is more neat, the plant height is 83 cm, lodging resistance is better, and the ripening is good. Spicle spindle-shaped, long-awned, white-shelled, red-grained, kernels oval, semi-hard-silty, full. The number of ears is 33.0 million/mu, the number of grains per ear is 38.9, and the weight of thousand grains is 38.8 g.
YJM586	Yangzhou Yangzijiang Seed Industry Co., Ltd., Yangzhou, Jiangsu, China; Henan Universtiy	Selected from Shenhxuan 3 variants	This variety belongs to spring medium-ripe wheat variety. The seedlings were semi-erect, the plant type was loose, and the tillering ability and panicle number were medium. Long awn, white shell, red grain, spindle-shaped, hard grain. The average result of district trial: the whole growth period was 209.1 days. Average plant height is 77.4 cm. Effective ear per mu is 331,000, the number of grains per ear is 39, 1000-grain weight is 40.8 g.
YM16	Agricultural Science Institute of Jiangsu Lixiahe District, Yangzhou, China	Yang 91F138/ Yang 90-30	A spring medium ripe wheat variety. Long-awned, white-shelled, spindle type ear, red-skinned, hard grain. The seedlings are erect and loosely shaped. The plant height is about 90 cm. The number of effective ears is about 260,000 per mu, and number of grains is about 38 grains per ear, and the 1000-grain weight is about 37 g.
YM33	Agricultural Science Institute of Jiangsu Lixiahe District, Yangzhou, China	Sumai 6/97G59//Yangmai 18	A spring variety, the whole growth period is 201.3 days. The seedlings are semi-creeping, the leaves are wide and short, the leaf color is dark green, and the tillering ability is medium. The plant height is 84.3 cm, the plant type is compact, lodging resistance is medium. The uniformity is good, the ear layer is neat, and the

			maturity is good. Spindles, long awns, red grains, silty grains, good fullness. The number of ears in mu is 318,000, the number of grains per ear is 39.4, and the 1000-grain weight is 43.3 g.
RH520	Jiangsu Ruihua Agricultural Technology Co., Ltd., Suqian, Jiangsu, China	Zhengzhou 891/Qianfeng 1	A semi-winter medium ripe variety, the whole growth period of 227 days. The seedlings are creeping, strong seedling strength, narrow and curly leaves, dark green leaves, winter cold resistance is good, the tillering ability is strong before winter, and the percentage of effective tillers is average. The spring jointing is late, the polarization is fast, and the ability of resistance to cold spring is medium. Late high temperature resistance is general, mature phase is general. The plant height is 87 cm, the stem is waxy and heavy, the stem is of average elasticity and lodging resistance. The plant type is slightly loose, the flag leaf is narrow and long, and the spike layer is thick. Spindles, long awns, white shell, white grain, keratin, medium fullness, low black germ rate. The number of panicle in mu was 422,000, the number of grains per panicle was 31.1, and the 1000-grain weight was 40.2 g.
ZM15	Jiangsu Ruihua Agricultural Technology Co., Ltd., Suqian, Jiangsu, China; Zhenjiang Institute of Agricultural Sciences in Hilly Area, Zhengjiang, Jiangsu, China	Ning 04126//(Yangmai 158/ok94p549-2e)^2	A spring medium ripe wheat variety. The seedlings are upright, with dark leaf color and medium tillering ability. The plant type is loose, lodging resistance is better. Ear layer is neat, ripeness is better. Spindles, long awns, white shells, red grains, hard seeds. The plant height was 82.6 cm, the number of effective ears was 312000 per mu, the number of grains per panicle was 37.5, and the 1000-grain weight was 45.9 g.
YM23	Jiangsu Kingearth Seed Co. Ltd., Yangzhou, China	Yangmai 16/Yangfu 93-11	A spring variety with a whole growth period of 202 days. The seedlings are semi-erect, with strong tillering, green leaves and vigorous growth. The plant is 84 cm tall, compact, and the flag leaves are raised upward. Ear layer is more orderly, ripeness is better. Spindles, long awns, white shells, red grains, seeds oval, hard, more full. The average number of ears per mu is 324,000, the number of grains per ear is 39.4, and the 1000-grain weight is 37.7 g.
STM1	Jiangsu Hongqi Seed Industry Co., Ltd., Taizhou, Jiangsu, China	Zhengmai 8329/ Yannong 19	The seedling is semi-creeping, with narrow leaves, dark green leaf color, semi-loose plant type, high tillering rate, plant height of about 87cm, compact plant type, beautiful appearance and good stalk elasticity.
XM44	Xuzhou Institute of Agricultural Sciences of the Xuhuai District of Jiangsu Province, Xuzhou, China	Xumai 7048/Xumai 32	A weak spring variety with a whole growth period of 228.2 days. The seedlings are semi-creeping, narrow leaves, green leaves, medium tillering ability. The plant height is 85.1 cm, the plant type is loose, lodging resistance is good, the uniformity is good, the ear layer is neat, the ripening phase is medium. Ear rectangle, long awn, white grain, kernel hard, more full. The number of ears in mu is 422,000, the number of grains per ear is 34.3, and the 1000-grain weight is 44.9 g.
HM35	Huaiyin Institute of Agricultural Sciences of the Xuhuai District of Jiangsu	Zhoumai 13/Xinmai 9	A semi-winter medium ripe variety, the whole growth period is 230 days. The seedlings are semi-creeping, growing well, the leaves are fine and curly, the leaves are thick and green, and the cold resistance is good in winter. The tillering power is strong and the tillering rate is low. The early spring jointing is faster, the polarization is slow, and the ability to resist cold spring is medium. The late high temperature resistance is

	Province, Huaian 223001, China		better, the grouting is fast, and the maturity is good. The plant height is 87 cm, the plant type is loose, lodging resistance is medium. The ear layer is thick, the flag leaves are short and upthrust, and the stem and leaves are waxy and heavy. Spindle-shaped, spindle-length thin, long awns, white shell, white grain, half-keratin grain, better fullness, slightly higher black embryo rate. The average number of ears per mu is 395,000, the number of grains per ear is 35.5, and the 1000-grain weight is 42.6 g.
HM20	Huaiyin Institute of Agricultural Sciences of the Xuhuai District of Jiangsu Province, Huaian 223001, China	Yumai 13/ Lumai 14	A mid-winter wheat variety. Spindle-type spike, white keratin, semi-crawling seedlings, deep leaf color, strong tillering, more panicle number, compact plant type, good stem strength, small blade and ascending, slow growth in early stage, accelerated growth rate after jointing, plant height about 85 cm; The number of panicle per mu is about 380,000, the number of grains per panicle is about 33, the thousand grain weight is about 42 g.
HM168	Anhui Lvyi Seed Industry Co., Ltd., Hefei, Anhui, China; Jiangsu Huanghuai Seed Industry Co., Ltd., Suqian, Jiangsu, China	Huaimai 33/HA68	A semi-winter variety. The whole growth period was 223.8 days. The seedlings are semi-creeping, green leaves, strong growth, short leaves, strong tillering, and average panicle number. The plant height is 85.7cm, the plant type is semi-compact, the flag leaf is uprushed, the stalk elasticity is good, the ear layer is neat, the head size is uniform. Rectangular ear, long awns, white shell, white grain, half horny, full grain. The average number of ears per mu is 415,000, the number of grains per ear is 38.6, and the 1000-grain weight is 42.2 g.
XM35	Xuzhou Institute of Agricultural Sciences of the Xuhuai District of Jiangsu Province, Xuzhou, China	Xinmai 93119/Zhoumai 16	A semi-winter variety, the whole growth period is 226 days. The seedlings are half creeping, the leaves are wide and short and erect, the tillering ability is strong, and the resistance to cold spring is medium. The plant height is 82.2 cm, the plant type is compact, the stem is thick, the elasticity is general, lodging resistance is general. The flag leaves are short and wide, uprushed, ear leaves are in the same layer, ear layers are neat, and ripening is relatively good. Spindle-shaped, white shell, long awns, white grain, kernels horny, medium fullness. The number of ears is 427,000 per mu, the number of grains per ear is 35.8, and the 1000-grain weight is 41.6 g.
RHM502	Jiangsu Ruihua Agricultural Technology Co. Ltd., Suqian, China	Zhengzhou 891/Qianfeng 1	A semi-winter variety, the whole growth period is 231.4 days, the seedlings is semi-creeping, narrow short leaves, dark green leaf color, strong tillering ability. The plant height is 84.6 cm, the plant type is compact, lodging resistance is better, uniformity is better, ear layer is more neat. Spindles, long awns, white grains, hard grains, fullness. The number of ears is 414,000 per mu, the number of grains per ear is 35.9, and the 1000-grain weight is 42.2 g.
HM17	Huaiyin Institute of Agricultural Sciences of the Xuhuai District of Jiangsu Province, Huaian, China	82057/ Xuzhou 7471	A weak spring variety, the whole growth period is 225 days. Plant height is 90 cm, long awns, white shell, white grain. loose plant type, strong tillering ability, good ripening.
HM16	Huaiyin Institute of Agricultural Sciences of the		A weak spring, multi-ear type, medium early maturing variety. The seedlings are half creeping, seedling growth is strong, tillering ability is medium, and the plant height is about 90 cm. Long awn, white shell, white grain, keratin, spindle spike, with about 29 grains per ear and about 38 g of 1,000 grains weight.

	Xuhuai District of Jiangsu Province, Huaian, China		
HY66	Anhui New Century Agriculture Co., Ltd., Fuyang, Anhui, China	Luyuan 502/ 9024	A semi-winter medium-ripe variety, the growth period is about 235 days. The seedlings are semi-creeping, the leaves are green, and the tillering ability is strong. The adult plant type is relatively compact, the plant height is about 71 cm. The number of ears in mu is about 420,000. Spiniform, long-awned, white shell, white grain, semi-hard, full grain. The number of grains per spike was 32.8, and the weight of thousand grains was 42.7 g.
HY1722	Anhui New Century Agriculture Co., Ltd., Fuyang, Anhui, China; Siyou Xu, Mingfang Liu, Dongxue Yang, Bin Wu, Fei Ma, Jiangpeng Hong, Dengcai Zhang, Huafeng Yan, Fei Ma, Jiangpeng Hong, Dengcai Zhang, Huafeng Yan, Xiaoling Ma, Zhiyong Hu	Jimai 22/ Han 6172	A semi-winter. The whole growth period is 225.2 days. The seedlings are creeping, slender leaves, dark green leaves, and strong tillering ability. The plant height is 80.5 cm, the plant type is compact, lodging resistance is good, uniformity is good, ear layer is neat, ripe phase. Spindles, long awns, white grains, hard, full grains. The number of ears in mu is 425,000, the number of grains per ear is 31.1, and the 1000-grain weight is 47.2 g.
WM203	Bozhou Institute of Agricultural Sciences, Bozhou, Anhui, China	Huaimai 20 / Aikang 58	A semi-winter variety. The whole growth period is 211.5 days. The seedlings are half creeping, the leaves are light green and wide, the seedlings grow strongly, and the tiller yield is average. Spindle-type, white, silty grain, full degree. The number of ears in mu is 408,000, the number of grains per ear is 45.3, and the 1000-grain weight is 45.1 g.
WY16	Bozhou Institute of Agricultural Sciences, Bozhou, Anhui, China	Zhoumai 27///Zhoumai 16/Jimai 22//Zhoumai 16	A semi-winter variety. The whole growth period is 211.7 days. The seedling is half creeping, the leaf color is light green, the seedling growth is strong, the tillering ability is general, the heading rate is high. The plant height is 82.6cm, the plant type is compact, the flag leaves are tilted upward, the stem wax powder is heavier, the ear layer is neat, and the ripening is good. Rectangular ear, long awns, white shell, white kernels, kernels full. The number of ears in mu is 402,000, the number of grains per ear is 35.9, and the 1000-grain weight is 43.3 g.
XSJ999	Anhui New Century Agriculture Co., Ltd., Fuyang, Anhui, China; Tai'an Academy of Agricultural Sciences, Tai'an, China	Yannong 999/Xuzhou 856	A semi-winter variety. The whole growth period is 224.1 days. The seedlings were semi-creeping, with dark green and wide leaves, poor tillering ability and high heading rate. The plant height is 89.3cm, the plant type is compact, the flag leaves are wide and long, the ear layer is neat, the stem wax is light, and the ripening phase is average. The ear is rectangular, long awning, the kernel is white, half horny and full. The number of ears in mu is 393,000, the number of grains per ear is 37.2, and the 1000-grain weight is 46.1 g.

ZYGK1	Anhui New Century Agriculture Co., Ltd., Fuyang, Anhui, China; Kai Ding, Yuwei Yang, Xiujuan Wang, Dongxue Yang, Xiangxiang Cao, Dahu Shang, Zhenghuan Yin, Binghong Cao, Zhihong Xu, Shaojun Ren	Huaimai 22/Yannong 0428	A semi-winter variety. The whole growth period is 224.6 days. The seedlings are creeping, slender leaves, dark green leaves, and strong tillering ability. The plant height is 84.5 cm, the plant type is compact, lodging resistance is better, the uniformity is good, the ear layer is neat, and the ripening is good. Spindles, long awns, white grains, hard grains. The number of ears in mu is 412,000, the number of grains per ear is 33.5, and the 1000-grain weight is 47.9 grams.
YH6	Anhui Gushen Seed Industry Co., Ltd.. Bozhou, Anhui, China	Ningmai 9/ R9088	A spring variety, the whole growth period is 202.1 days. The seedlings were semi-erect and the tiller yield was average. Good uniformity, average plant height is 79.8 cm. Plant type is loose, spindle type ear, white shell, long awn, red grain, kernels half horny. The number of ears in mu is 320,000, the number of grains per ear is 40.2, and the 1000-grain weight is 38.8 g.
JM52	Crop Research Institute, Shandong Academy of Agricultural Sciences, Jinan, Shandong, China	Shannong 22/ Jimai 22	A semi-winter variety , the whole growth period is 237.7 days. The seedlings are semi-creeping, with wide leaves, dark green leaf color and medium tillering ability. The plant height is 77.3 cm, the plant type is loose, lodging resistance is medium, the uniformity is good, the ear layer is neat, and the ripening is good. Ear rectangle, long awn, white grain, hard, full grain. The number of ears in mu is 379,000, the number of grains per ear is 30.9, and the 1000-grain weight is 43.0 g.
JM54	Crop Research Institute, Shandong Academy of Agricultural Sciences, Jinan, Shandong, China	Xitainong 19/Jimai 22	A semi-winter variety, the seedling is semi-creeping, plant type is semi-compact, dark green leaf color, slender leaves, lodging resistance, ripening phase. Long awn, white shell, white grain, hard grain. The growth period was 226 days, the plant height was 82.51 cm, the number of effective ears was 413,300 per mu. The spike was rectangular, the number of grains per spike was 36.4, the 1000-grain weight was 41.55 g, and the bulk weight was 789.86 g L^{-1}.
JM60	Crop Research Institute, Shandong Academy of Agricultural Sciences, Jinan, Shandong, China	Ji 037042/Jimai 20	A semi-winter variety, the seedling is semi-upright, plant shape is semi-compact, deep leaf color, leaf upthrust, lodging resistance, good ripening. The growth period of plants is 229 days, plant height is 74.8 cm. Long awn, white shell, white grain, hard grain. The number of ears in mu is 385,000, the number of grains per ear is 35.4, and the 1000-grain weight is 41.5 g.
JM262	Crop Research Institute, Shandong Academy of Agricultural Sciences, Jinan, Shandong, China	Linmai 2/ Yannong 19	A winter variety, the seedling is semi-erect. The plant type is semi-compact, the flag leaves are broad and downcast, and it is more resistant to lodging and the ripening phase is medium. Long awn, white shell, white grain, full grain, silty. The plant height was 67.2 cm, the maximum tiller in mu was 748,000, the effective ear in mu was 327,000. The panicle was rectangular, the number of grains per panicle was 37.5, the 1000-grain weight was 44.7 g.
JM379	Crop Research	Derived from the physical and	A semi-winter variety, the whole growth period is 234.1 days. The seedlings are semi-creeping, slender leaves, dark green leaf color, strong tillering ability. The plant height is 84.5 cm, the plant type is compact,

	Institute, Shandong Academy of Agricultural Sciences, Jinan, Shandong, China	chemical mutagenesis of Jimai 06037	lodging resistance is better, the uniformity is good, the ear layer is neat, and the ripening is good. Ear rectangle, long awn, white grain, hard grain, fuller degree. The number of ears in mu is 442,000, the number of grains per ear is 37.8, and the 1000-grain weight is 44.1 g.
YN301	Yantai Academy of Agricultural Science, Yantai, Shandong, China	Jimai 22 × Yan 1201	A winter variety, the seedling is semi-creeping, plant type is semi-compact, dark green leaves, lodging resistance, medium ripening phase. The average growth period of two years was 235 days. Long awn, white shell, white grain, hard grain. The plant height was 79.1 cm, the maximum tiller in mu was 998,000, the effective ear in mu was 413,000, and the tiller yield was 41.9%. The spike was rectangular, the number of grains per spike was 38.0, the 1000-grain weight was 44.6 g.
YN1212	Yantai Academy of Agricultural Science, Yantai, Shandong, China	Yan 5072/ Shi 94-5300	A conventional variety, semi-winter, semi-creeping seedlings, semi-compact plant type, dark green leaf color, upthrust leaves, good lodging resistance and good ripening. Long awns, white shells, white grains, semi-hard seeds. The whole growth period was 232.1 days, the plant height was 76.2 cm, the maximum tiller was 962,000 mu, the effective ear was 415,000 mu, and the tiller yield was 43.7%. Clav-shaped spike, grain number per spike is 38.9, 1000-grain weight is 43.7 g.
YN999	Yantai Academy of Agricultural Science, Yantai, Shandong, China	Yanhangxuan 2/Lin 9511F1//Yan BLU14-15	A semi-winter variety, the whole growth period is 227 day. The seedlings are creeping, strong seedling potential, narrow leaf roll, dark green leaf color, winter cold resistance is better. The tillering ability is strong, and the tillering yield is medium. In spring, the jointing is slow, the heading is late, and the ability to withstand spring cold is better. In the later stage, the root system had strong vitality, average resistance to high temperature, and better ripening. The plant height is 88 cm, the stem elasticity is medium, lodging resistance is average. The plant type is compact and the stem wax layer is thick. Flag leaf wide and long, slightly draped, ear layer thick. Ear rectangle, slender ear, spikelet dense, long awn, white shell, white grain, keratin, medium fullness. The number of ears per mu is 400,000, the number of grains per ear is 33.8, and the 1000-grain weight is 44.2 g.
SN41	Shandong Agricultural University	Tainong 18/ Linmai 6	A semi-winter variety, the whole growth period is 227.8 day. The seedlings are nearly creeping, the leaves are wide and short, the leaf color is dark green, and the tillering ability is strong. The plant height is 78.3 cm, the plant type is compact, lodging resistance is medium. The ear layer is neat and the ripening phase is normal. Spindles, long awns, white grains, kernels half horny, medium fullness. The number of ears in mu is 432,000, the number of grains per ear is 31.9, and the 1000-grain weight is 42.4 g.
ZXM998	Hebei Bogenesis Technology Co., Ltd., Handan, Hebei, China; Hebei Manxin Agricultural Technology Co., Ltd., China	Laizhou 137/ Zhongxin 5072	A semi-winter variety, the whole growth period is 221.5 days. The seedlings are half creeping, with wide and long leaves, dark green leaf color, strong tillering ability and medium heading rate. The plant height is 72.4 cm, the plant type is compact, lodging resistance is better. The ear layer is neat, the flag leaf is wide, raised, and ripe. Ear rectangle, long awn, white shell, white grain, kernels half horny, good fullness.

TM178	Henan Tiancun Seed Agricultural Science, Co., Ltd., Zhengzhou, Henan, China	Zheng 366/BainongAK58 //Zhoumai 22	A semi-winter variety, the whole growth period is 219.1 ~ 229.7 days. The seedlings are upright, the leaves are dark green, the seedling strength is strong, the winter cold resistance is good, the tillering ability is strong, the heading rate is high. Spring rise early jointing, polarization fast, early heading, resistance to spring cold medium. The plant height is 76.4 ~ 82.4 cm, the plant type is compact, lodging resistance is better. The flag leaf is short, the ear section is long, the ear layer is more neat, and the ripening is good. Ear rectangle, long awn, white shell, white grain, kernels half horny, full degree is good. The number of ear per mu is 398,000, the number of grains per ear is 36.6, and the 1000-grain weight is 46.4 g.
SM132	Suiping Experimental Station of Agricultural Sciences, Zhumadian, Henan, China; Henan Fengge Agricultural Technology Co., Ltd., Zhumadian, Henan, China	Yunong 416/ XNS16-2	A semi-winter variety, the whole growth period of was 219.3 ~ 227.5 days. The seedlings are semi-erect, the leaves are dark green, the seedling potential is general, the tillering ability is strong, and the percentage of effective tillers is high. Plant height is 70.8 ~ 78.6 cm, plant type is compact, lodging resistance medium. The lower ear section is shorter, the ear layer is more orderly, and the ripening is good. Ear rectangle, short awns, white shell, white grain, keratin, fullness is good. The number of ears in mu is 422,000, the number of grains per ear is 32.5, and the 1000-grain weight is 46.4 g.
SD29	Henan Shangdao Seed Industry Co., Ltd., Henan, China; Xianxin Chen, Haisheng Zhang, Ming Chen	Xinong 979/ Xinmai 26	A semi-winter variety, the whole growth period was 224.1 days. The seedlings are semi-creeping, with slender leaves, dark green leaves and strong tillering ability. The plant height is 75.2 cm, the plant type is compact, lodging resistance is good, the uniformity is good, the ear layer is neat, and the ripening is good. Ear rectangle, long awn, white grain, hard, full grain. The number of ears in mu is 402,000, the number of grains per ear is 37.5, and the 1000-grain weight is 46.8 g.
ZM136	Institute of Wheat Research, Henan Academy of Agricultural Sciences, Zhengzhou, Henan,China	Aikang 58/ Jimai 22	A semi-winter variety, the whole growth period is 225 days. The seedlings are semi-creeping, narrow and short leaves, yellow-green leaf color, and medium tillering ability. The plant height is 76 cm, the plant type is compact, lodging resistance is better. The flag leaves are raised, the uniformity is good, the ear layer is thick, the ripening is good. Spindles, long awns, white shells, white grains, kernels half horny, full degree is good. The number of ears per mu is 410,000, the number of grains per ear is 31.6, and the weight of thousand grains is 45.1 g.
ZM1860	Institute of Wheat Research, Henan Academy of Agricultural Sciences, Zhengzhou, Henan,China	Zhoumai 22/Zhengmai 1410//Zhengmai 0856	A semi-winter variety, the whole growth period is 232 days. The seedlings are semi-creeping, narrow leaves, light green leaves, and strong tillering ability. The plant height is 80 cm, the plant type is slightly loose, lodging resistance is better. The flag leaves are raised, the uniformity is good, the ear layer is neat, and the ripening is relatively good. Ear oval, short awns, white shell, white grain, keratin, good fullness. The number of ears in mu is 379,000, the number of grains per ear is 34.9, and the 1000-grain weight is 48.5 g.
RH116	Zhigang Sun, Qian Meng, Zhengping Fu, Pengyun Chen, Weiqiang Dong, Buwen Mu, Yinjuan Hou	Zhengmai 366/Shanmai 159//Zhoumai 22	A semi-winter variety, the whole growth period is 223.2 days. The seedlings were half creeping, with good growth and strong tillering ability. The plant height is 73 cm, the plant type is semi-loose, lodging resistance is better. The uniformity is good, the ear layer is neat, and the maturity is good. Ear nearly rectangular, long awn, white grain, kernels half horny, good fullness. The number of ears in mu is 383,000, the number of grains per ear is 34.9, and the 1000-grain weight is 42.9 g.

WL169	Shaanxi Yangling Weilong Agricultural Technology Co., Ltd., Yangling, shaanxi, China	Shanmai 94/ Xinong 822	A semi-winter variety, the total growth period is 216 days. The seedlings are semi-creeping, narrow leaves, yellow-green leaf color, and medium tillering ability. The plant height is 74.2 cm, the plant type is compact, lodging resistance is better. The uniformity is good, the ear layer is neat, and the ripening is better. Ear shape nearly rectangular, long awn, white grain, keratin, good fullness. The number of ears in mu is 40.0 million, the number of grains per ear is 34.8, and the 1000-grain weight is 40.6 g.
LM30	Jiangsu Tianlong Science & Technology Co., Ltd., Suzhou, Jiangsu, China	Zhenmai 168/ Huamai 5	A spring wheat variety, the seedling is semi-erect with medium leaf color and medium tillering ability. The plant type is loose and lodging resistance is better. Ear layer is more orderly, ripeness is better. Spindles, long awns, white shells, red grains, kernels semi-horny. Average result of district trial: the whole growth period is 202.5 days. Plant height 81.5 cm, effective ear per mu 302,000, 41.2 grains per ear, 1000-grain weight 43.9 g.
